# Synthetic Strategies for Activity‐Based Probes to Decode Ubiquitin‐Like Modifiers

**DOI:** 10.1002/chem.202503597

**Published:** 2026-02-23

**Authors:** Saibal Chanda, Alan Pham, SreeNidhi Karnati, Natalia Soto Rodriguez, Alyssa Toner, Wenshe Ray Liu

**Affiliations:** ^1^ Department of Biochemistry and Biophysics, College of Agriculture and Life Sciences Texas A&M University USA; ^2^ Texas A&M Drug Discovery Center and Department of Chemistry, College of Arts and Sciences Texas A&M University USA; ^3^ Institute of Biosciences and Technology and Department of Translational Medical Sciences, School of Medicine Texas A&M University Houston USA; ^4^ Department of Cell Biology and Genetics, School of Medicine Texas A&M University USA; ^5^ Department of Pharmaceutical Sciences, Irma Lerma Rangel College of Pharmacy Texas A&M University USA

**Keywords:** activity‐based probe, activity‐based protein profiling, chemical ligation, ubiquitin, ubiquitin‐like protein

## Abstract

Ubiquitin‐like proteins (Ubls) such as SUMO, NEDD8, ISG15, URM1, UFM1, FAT10, ATG8/ATG12, and FUBI are essential regulators of cellular homeostasis, controlling processes from protein stability and trafficking to immune signaling and autophagy. Their conjugation–deconjugation cycles are mediated by cascades of E1, E2, and E3 enzymes and reversed by Ubl‐specific proteases (ULPs), many of which are cysteine‐dependent. Deciphering these dynamic and reversible pathways requires tools that directly capture the active forms of these enzymes. Activity‐based probes (ABPs) have become indispensable for this task, providing covalent, mechanism‐based snapshots of enzymatic activity in complex systems. This review highlights chemistry‐centric strategies for the design and synthesis of Ubl‐targeting ABPs. We summarize synthetic and semisynthetic approaches that install electrophilic warheads onto Ubl backbones, methods for *C*‐terminal ligation (native chemical ligation, activated cysteine ligation, hydrazide chemistry), and strategies for incorporating reporter tags or bioorthogonal handles. Probe development is organized by target class, including Ubl isopeptidases, E1/E2 conjugating enzymes, and E3 ligases. Representative examples illustrate how chemical design choices are tailored for specific applications—ranging from live‐cell activity profiling to proteomic mapping and inhibitor discovery. Together, these methodologies establish a versatile chemical toolkit for dissecting Ubl biology, enabling the discovery of novel enzymes, the mapping of substrate networks, and the development of potential therapeutic modulators.

## Graphical abstract

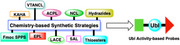

## 1 Introduction

**FIGURE 1:**
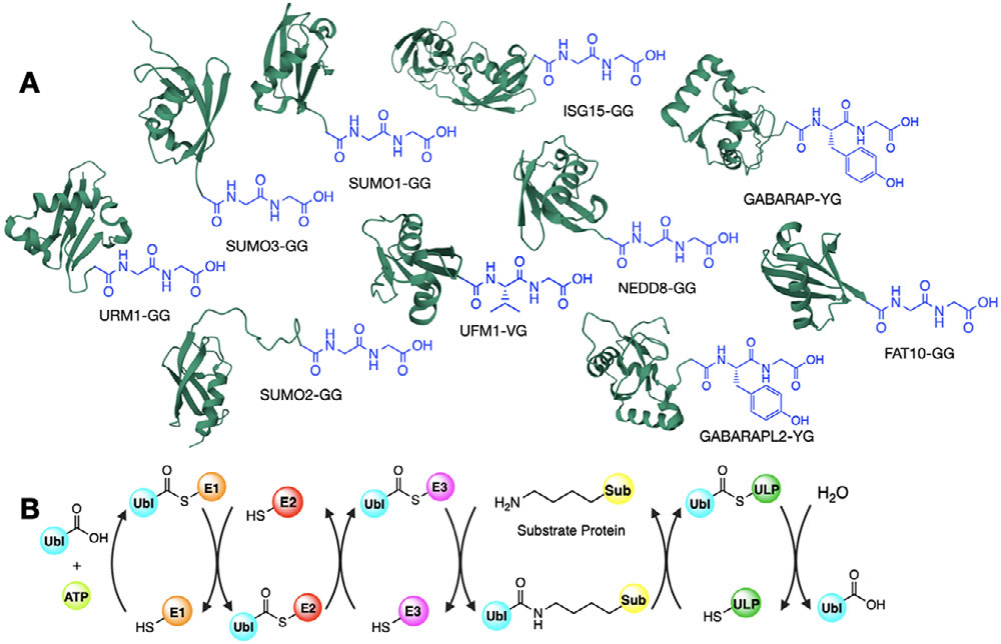
Structure of several Ubls and their conjugation/deconjugation cascade (A) Structure of several Ubls such as URM1 (PDB: 2QJL), SUMO1‐3 (PDB: 2N1V, 1WM3, 1U4A), ISG15 (PDB: 7S6P), NEDD8 (PDB: 1NDD), UFM1 (PDB: 5IA7), GABARAP (PDB: 1GNV), GABARAPL2 (PDB: 4CO7), FAT10 (PDB: 6GF1), and their terminal amino acids. (B) The E1–E2–E3 cascade attaches Ubls to target proteins through sequential thioester transfer: ATP‐dependent activation by E1, transthiolation to E2, and substrate ligation by E3. Ubl‐specific proteases (ULPs) reverse this modification. Each step provides an entry point for activity‐based probes to covalently trap active enzymes and enable functional profiling.

Ubiquitin and numerous ubiquitin‐like proteins are pivotal post‐translational modifiers that regulate protein homeostasis and a wide range of cellular processes (from protein degradation to signal transduction in pathways like cell cycle control, transcription, DNA repair, and apoptosis) [1, 2, 3, 4, 5]. As shown in Figure 1A, major Ubl family proteins in humans include the SUMO (Small Ubiquitin‐like Modifier) paralogs, NEDD8 (Neural Precursor Cell Expressed Developmentally Down‐regulated 8), ISG15 (Interferon‐stimulated Gene 15), URM1 (Ubiquitin‐related Modifier 1), UFM1 (Ubiquitin‐fold Modifier 1), FAT10 (HLA‐F‐adjacent Transcript 10), the autophagy‐related ATG8/LC3 (Autophagy‐related Protein 8/Microtubule‐associated proteins 1A/1B Light Chain 3A) and ATG12 (Autophagy‐related Protein 12) modifiers, and FUBI (Fau Ubiquitin‐like Protein) [6] Like ubiquitin, these modifiers are conjugated to target proteins via an enzymatic cascade of E1 activating enzymes, E2 conjugating enzymes, and E3 ligases; they are removed by dedicated isopeptidases (such as deubiquitinase enzymes for ubiquitin or SENP proteases for SUMO) to reverse the modification (Figure 1B) [6, 7]. These isopeptidases are often described as Ubl‐specific proteases (ULPs) [8]. Given the dynamic and reversible nature of ubiquitination and Ubl conjugation, tools that can capture and characterize the active forms of Ubl‐processing enzymes are invaluable for dissecting these pathways in cells and in vitro. Activity‐based probes (ABPs) have emerged as powerful chemical tools to meet this need, enabling direct profiling of enzyme activity in complex biological systems [9, 10, 11]. An ABP is typically a modified substrate analog designed to bind an enzyme's active site and then form a covalent bond with that enzyme, effectively “locking” the enzyme in the act of catalysis. Ubl‐derived ABPs generally feature a three‐part design: (1) a recognition element (often a full‐length ubiquitin or Ubl protein, or a peptide fragment thereof) that confers specificity by engaging the target enzyme; (2) a reactive electrophilic warhead in place of the native scissile bond, which reacts with a nucleophilic residue in the enzyme's active site; and (3) an optional reporter tag or handle for detecting and enriching labeled enzymes (such as a fluorophore for imaging, biotin or FLAG epitope for affinity capture, or a bioorthogonal group for click‐chemistry labeling). Upon interaction with an active enzyme, the probe undergoes what would normally be a hydrolysis or transfer reaction, but formation of an irreversible covalent adduct with the enzyme's catalytic residue [12, 13]. This mechanism yields a stable enzyme‐probe complex, allowing only actively engaged enzymes to be tagged. In turn, ABP labeling serves as a readout of enzyme activity (since inactive or transiently inactive enzymes do not react), enabling selective profiling of active enzyme populations rather than just total protein levels. ABPs have been widely applied to map deubiquitinase activities, to discover or identify previously unknown Ubl‐specific proteases, and even to screen for or characterize enzyme inhibitors in the ubiquitin/Ubl system [14, 15, 16]. For example, broad‐spectrum electrophilic probes based on ubiquitin and Ubls have revealed unexpected cross‐reactivities and new enzyme specificities, such as ubiquitin hydrolases that can also process NEDD8, ISG15, or FUBI, thereby uncovering proteases in Ubl pathways that were not apparent from sequence alone [17, 18, 19]. Crucially, many enzymes in ubiquitin and Ubl pathways are cysteine‐dependent (e.g., most deubiquitinases, as well as the active‐site cysteines in E1 and E2 enzymes and in certain HECT/RBR E3 ligases), which makes them especially amenable to targeting by electrophilic probes. The inherent nucleophilicity of these catalytic cysteine residues has been effectively exploited by ABPs, explaining the success of numerous probes in irreversibly capturing active ubiquitin/Ubl enzymes in cell extracts and live‐cell settings [15, 20, 21, 22, 23, 24, 25, 26].

This review surveys advanced chemical strategies for constructing activity‐based probes (ABPs) targeting ubiquitin‐like proteins (Ubls). We focus on synthetic and semisynthetic methodologies that enable site‐specific installation of electrophilic warheads at the Ubl *C*‐terminus. These approaches facilitate modular probe design and support the creation of covalent, structurally faithful Ubl analogs compatible with diverse profiling contexts. Emphasis is placed on the mechanistic rationale linking chemistry to function. Collectively, this chemical toolbox empowers deep functional interrogation of Ubl signaling networks through covalent capture, activity mapping, and inhibitor discovery.

## 2 Intein‐Mediated Semi‐Synthesis and Expressed Protein Ligation (EPL)

Intein‐based semisynthesis is a powerful method to obtain Ubls with reactive *C*‐termini suitable for warhead installation. The original method of Expressed Protein Ligation was developed from Native Chemical Ligation, as an alternative to, at the time, synthesizing proteins in a semisynthetic method as the production of recombinant thioester proteins was unavailable [27, 28]. In this approach, the Ubl of interest is expressed in *E. coli* as a fusion to a self‐splicing intein (often with a chitin‐binding domain for purification). A key mutation in the intein (e.g., *C*‐terminal Asn → Ala) prevents full splicing and allows an external nucleophile to trigger cleavage. The procedure involves several steps:

### 2.1 Expression of a Ubl‐Intein Fusion

The Ubl is cloned *N*‐terminal to an intein domain (e.g., Mxe GyrA intein) and expressed. The intein's *C*‐terminus is mutated to block standard peptide release. Cells are lysed, and the fusion protein is affinity‐purified (e.g., on chitin resin via the intein's chitin‐binding tag) [29, 30]. This permits stability until the reaction is desired, at which point the cleavage is induced.

### 2.2 Thiolate‐Induced Cleavage to Generate a Thioester

The resin‐bound fusion is treated with a thiol such as MESNa (mercaptoethanesulfonate) or DTT/cysteamine at neutral pH. This induces an N→S acyl shift at the Ubl‐intein junction and traps the Ubl as a *C*‐terminal thioester [31, 32]. Typically, a Ubl‐thioester is released in yields of a few milligrams per liter of culture (on the order of 4–5 mg/L for ubiquitin). For example, ubiquitin‐MESNa thioester (Ub‐MESNa) can be prepared in this way.

### 2.3 Warhead Installation by Aminolysis

**FIGURE 2:**
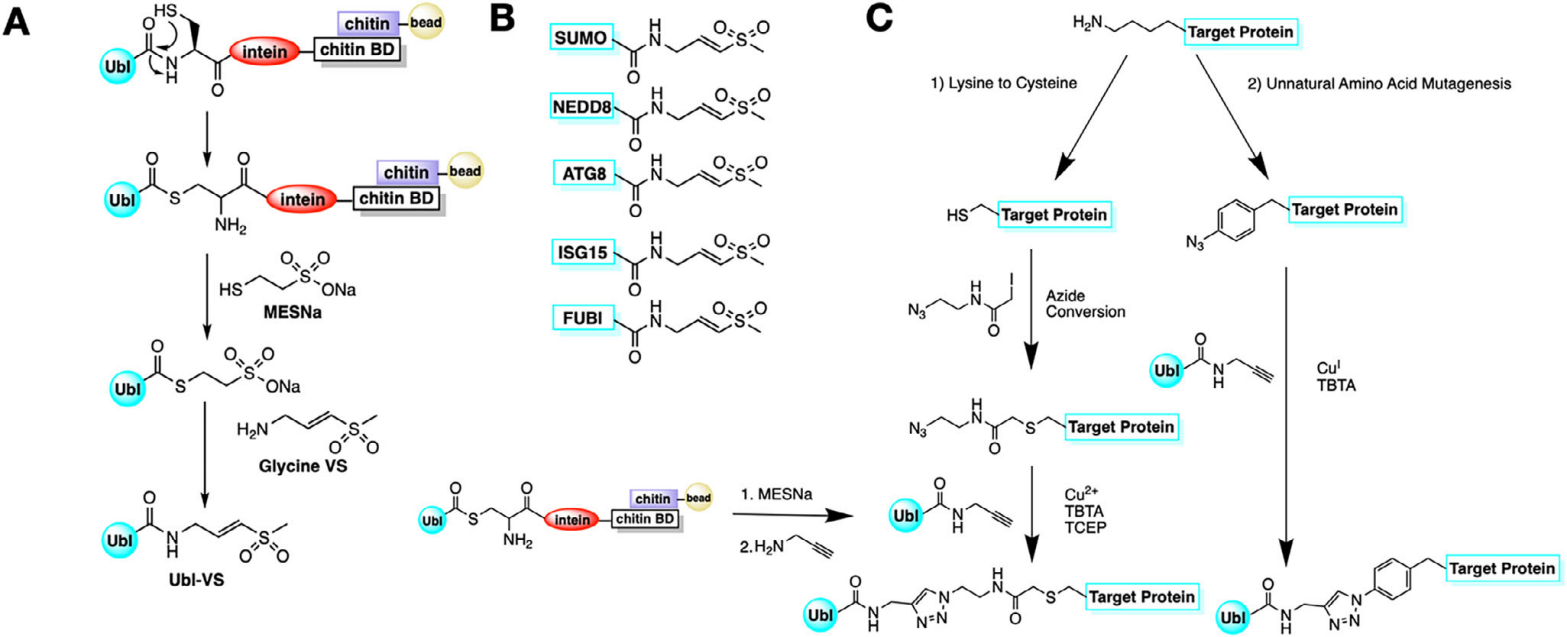
Intein‐based semi‐synthesis. (A) The expressed protein ligation on chitin resin and intein is converted to thioester using MESNa, followed by the installation of an electrophilic warhead using small molecule amines (e.g., glycine VS). (B) Vinyl sulfone activity‐based probes that have been synthesized using this intein‐mediated semi‐synthesis. (C) The usage of click chemistry‐based probes, which leverage intein semi‐synthesis to create unique bioconjugates. The creation of the conjugates can proceed through either a lysine to cysteine mutation or unnatural amino acid mutagenesis, which will allow for different conjugates depending on the desired result.

The Ubl thioester is then reacted with a warhead‐containing amine to form a new *C*‐terminal amide bond bearing the electrophile. This step is essentially a chemoselective ligation: the thiol‐thioester is displaced by the incoming amine (a mild aminolysis), as observed in Figure 2A. For instance, Hemelaar et al. expressed NEDD8, ISG15, and SUMO‐1 as thioesters and reacted them with glycine vinyl sulfone (VS) to attach a vinyl sulfone moiety [33, 34, 35]. The result was Ubl‐VS, for example, NEDD8‐VS, which covalently modifies cysteine proteases via Michael addition [36]. Similarly, reacting a Ubl thioester with propargylamine yields Ubl‐propargylamine [20]. Ubiquitin‐propargylamine (Ub‐PA) is obtained in a single step by thiolysis of the intein and aminolysis with propargylamine, producing a probe that reacts with active‐site cysteines to form a stable vinyl thioether adduct. This intein/EPL route can similarly produce vinyl methyl ester (VME) probes (e.g., Ub/Ubl‐VME) by aminolysis with an appropriate amine or alcohol derivative (details discussed under warheads below) [37]. A brief summary of the probes that have been generated is shown in Figure 2B.

### 2.4 Product Folding and Purification

Intein‐mediated reactions typically occur under mild, aqueous conditions that preserve the Ubl's folded state. Thus, the semisynthetic Ubl‐warhead is often obtained already folded and active. The product can be buffer‐exchanged to remove excess small‐molecule reagents and analyzed by MS. If needed, further purification is done by chromatography (e.g., HPLC or ion exchange) to isolate a homogeneous probe [38, 39]. In practice, affinity tags can also be incorporated (for example, expressing the Ubl with an *N*‐terminal hexahistidine or HA tag that remains after intein cleavage) to aid purification and serve as a detection handle. Some probes (like HA‐tagged Ub‐VME or fluorescent Ub probes) have been purified by Ni‐NTA or immunoaffinity methods, respectively.

### 2.5 Mechanistic Considerations

Intein semisynthesis leverages the intein's native protein splicing chemistry. The key is an N‐to‐S acyl transfer: a cysteine or serine at the Ubl's *C*‐terminus (part of the intein junction) forms a thioester with the Ubl's carbonyl, which is then captured by an external thiol [40]. This yields a stable thioester that can undergo a second acyl transfer when exposed to an amine (forming an amide bond). This chemoselective ligation occurs without perturbing other functional groups on the protein [41]. The intein method is notable for preserving protein integrity under conditions (pH ∼7, moderate temperature) that avoid unfolding, so the Ubl remains properly folded with its tertiary structure intact. Indeed, Bode and coworkers noted that even delicate Ubls like UFM1 retained their folded conformation through a hydrazide/thioester conversion and warhead attachment without side reactions on other residues.

### 2.6 Example—Ubl‐VS Probes

The first Ubl ABPs were prepared via intein‐EPL: Hemelaar et al. (Ovaa and Ploegh's groups) produced NEDD8, ISG15, and SUMO‐1 vinyl sulfone probes by this method [33]. The expressed Ubl‐intein was thiolyzed to give Ubl‐MESNa thioester, then treated with glycine‐vinyl sulfone to form a *C*‐terminal vinyl sulfonamide. These probes (e.g., SUMO1‐VS, NEDD8‐VS) specifically labeled their respective proteases (SENPs, NEDP1, etc.) via a conjugate addition of the active‐site Cys to the electrophilic double bond. Notably, the vinyl sulfone warhead reacts by Michael addition to yield a stable thioether linkage to the enzyme. The same strategy was later extended to other Ubls (e.g., FAT10‐VS, ATG8‐family VS probes) by preparing short Ubl *C*‐terminal peptide vinyl sulfones that could covalently modify proteases.

### 2.7 Purification and Availability

Semisynthetic Ubl probes can often be obtained at high purity by following the above protocol. After reaction, the probe is usually dialyzed or gel‐filtered to remove excess small molecules, and then analyzed by SDS‐PAGE and MS. If needed, HPLC purification yields an analytically pure product. Many standard ubiquitin ABPs prepared by intein methods have become commercially available. For example, HA‐tagged Ub‐VME and Ub‐VS, or fluorescent Ub‐PA probes, are sold by reagent companies. These are typically produced in bulk by expression and chemical ligation, then lyophilized for distribution. In contrast, more exotic probes (e.g., UFM1 or FAT10 ABPs) are usually prepared on a custom basis in research labs. Nonetheless, the intein‐EPL approach makes it feasible for any lab with molecular biology expertise to generate Ubl probes without needing full synthetic chemistry capability, bridging the gap between recombinant protein production and chemical modification.

### 2.8 Click Chemistry and Bioorthogonal Ligation

Leveraging the intein semi‐synthesis pathway, click chemistry can be used to prepare conjugates of Ubls to target substrate proteins. One of the key reactions used in click chemistry is Copper‐catalyzed azide‐alkyne cycloaddition, or CuAAC [42]. Reported by Weikart and coworkers, CuAAC has been able to be used to link SUMO and a RING finger protein [43]. Starting with a SUMO that has had two glycines removed to later mimic the native isopeptide bond length and was fused to an intein, they were able to generate a propargylamine‐probe type construct (although it is also safe to assume this same process could be replicated with other ligation methods mentioned in this review). Concurrently, their target protein's acceptor lysine was mutated into a cysteine to act as a selective chemical handle. This mutant was then incubated with iodoacetamide ethyl azide, which gave it azide functionality. Then, both the propargyl terminal protein and the azide‐functioning mutant were incubated together with copper, allowing for the formation of a new synthetic conjugate. An alternative to the mutation to cysteine involves the mutation of the lysine acceptor handle to an unnatural amino acid, allowing for the usage of this click chemistry even when other cysteines are present, thereby removing the possibility of a disulfide bond [44]. These parallel techniques are displayed in Figure 2C. These synthetic conjugates provide modifications of Ubls into substrate proteins, which would otherwise not have catalytic cysteines, and allow for easier synthesis of conjugates that are normally difficult to obtain.

## 3 Native Chemical Ligation and Auxiliary‐Based Peptide Ligation

Native chemical ligation (NCL) is a cornerstone of protein total synthesis, allowing two unprotected peptide segments to be joined by a peptide bond [45]. In NCL, a peptide with a *C*‐terminal thioester reacts with another peptide bearing an *N*‐terminal cysteine to form a native amide linkage at the ligation site. Applied to Ubls, NCL enables assembling full‐length Ubls from smaller synthetic fragments and can be used to introduce warheads, tags, or other modifications at the junctions. Key features of NCL in Ubl probe synthesis include:

### 3.1 Segment Assembly of Ubls

**FIGURE 3:**
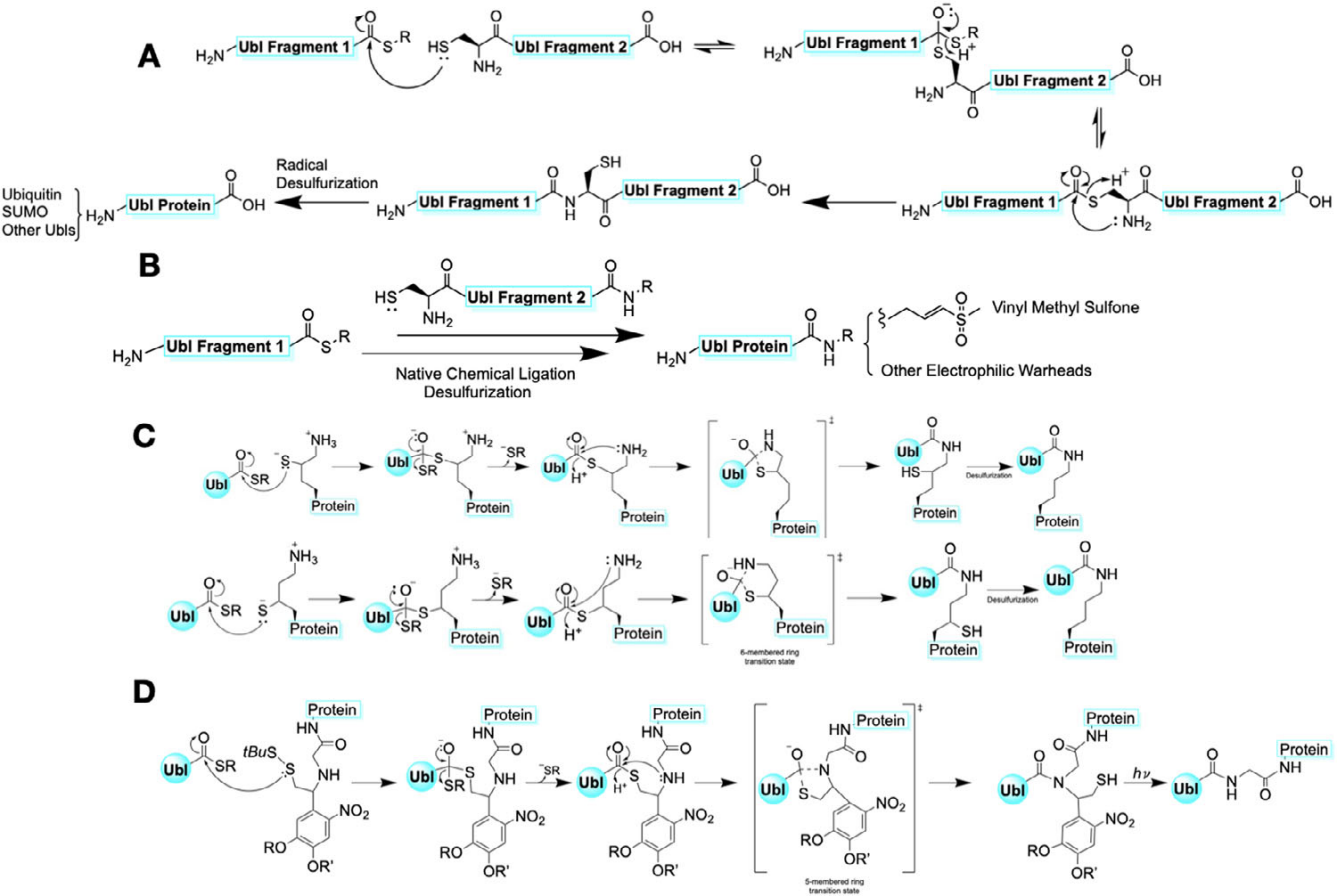
Native chemical ligation and its applications in the synthesis of Ubls and their ABPs. (A) The general reaction mechanism of native chemical ligation applied to the synthesis of a Ubl using a cysteine residue present on the *N*‐terminus of a fragment to ligate onto the *C*‐terminus of another fragment, which has been functionalized with a thioester bond. (B) The usage of native chemical ligation to perform the synthesis of activity‐based probes with an electrophilic warhead at the *C*‐terminus. (C) Native chemical ligation is applied in the synthesis of bioconjugates through the usage of δ‐mercaptolysine and γ‐mercaptolysine building blocks, proceeding through different transition states that inevitably lead to the same product. (D) The usage of a photocleavable glycine auxiliary to mimic a native lysine linkage using a similar mechanism to the mercaptolysine auxiliary.

Ubiquitin (76 aa) and many Ubls exceed the length easily made by one SPPS run. Therefore, they are divided into two or more peptide segments for synthesis. For example, Brik et al. synthesized Ub in two pieces: residues 1–45 (with a *C*‐terminal thioester) and residues 46–76 (with an *N*‐terminal Cys at position 46). Ligation of these two segments in aqueous buffer yielded full‐length Ub with a Cys at position 46, which was later converted to Ala by radical desulfurization to restore the native sequence [46]. This approach produced an Ub‐thioester ready for further use (e.g., to ligate onto another peptide or to act as a donor in forming ubiquitinated substrates). Similarly, a two‐segment strategy can produce a Ub *C*‐terminal hydrazide (by incorporating a *C*‐terminal hydrazide in one segment) as an alternative handle. Figure 3A depicts the native chemical ligation of Ubl fragments into a fully‐fledged Ubl. SUMO proteins (∼90 aa) and others have also been prepared by segment ligation; Ovaa's group even reported direct SPPS of a 92‐residue SUMO‐2 by using special coupling cocktails and aggregation breakers [47].

### 3.2 Warhead Introduction via a Synthetic Peptide

NCL is particularly useful for installing electrophilic warheads that are easier to handle on small peptide fragments. For instance, one can synthesize a short *C*‐terminal peptide (e.g., the *C*‐terminus of a Ubl plus an extra glycine or linker bearing the warhead) and ligate it to a large *N*‐terminal fragment of the Ubl. Figure 3B shows a general reaction mechanism for the introduction of a warhead through native chemical ligation of a synthetic peptide. As an example, one could prepare a peptide comprising ubiquitin residues 64–76 with an *N*‐terminal Cys (for ligation) and a *C*‐terminal vinyl sulfone moiety. Ligation of this to Ub(1–63)‐thioester would yield full‐length Ub‐VS entirely by chemical synthesis. This strategy has been used to create di‐ubiquitin or di‐Ubl probes where a warhead is placed at an isopeptide linkage site (see below). Because NCL tolerates a wide range of functional groups (performed in mildly reducing conditions, pH∼7 in buffers with thiol additives), delicate electrophilic warheads can survive the ligation step. After ligation, any auxiliary atoms (like a ligation cysteine that is not native) can often be removed by desulfurization to regenerate a native residue.

### 3.3 Isopeptide Bond Formation via Auxiliaries

Ubl conjugates (e.g., ubiquitin chains or Ubl‐substrate fusions) involve linking the Ubl's *C*‐terminus to a lysine sidechain on another protein. Reproducing this isopeptide in a synthetic context requires special tactics since standard NCL only joins termini. Auxiliary‐based ligation methods solve this by temporarily converting a Lys into a cysteine‐equivalent. One approach is attaching a thiol‐bearing auxiliary to the Lys's side chain to create a ligation handle. For example, as shown in Figure 3D, a photolabile thioglycine auxiliary can be installed on a Lys, which then undergoes NCL with a Ubl‐thioester, forming a ligation at the Lys position; subsequent UV irradiation removes the auxiliary, restoring a native Lys linkage [48]. Alternatively, a “mercaptolysine” (lysine analog with a thiol in the side chain) can be incorporated into the peptide sequence. After ligation through the mercaptolysine's thiol and a Ubl‐thioester, a chemical desulfurization converts the linkage to a normal Lys isopeptide bond [49]. Brik and coworkers developed δ‐mercaptolysine and γ‐mercaptolysine building blocks for this purpose [50]. Figure 3C shows these auxiliary strategies, which enable assembly of poly‐Ubl chains and conjugates that are indistinguishable from native linkages once the auxiliary is removed. They are extremely useful for making specific ubiquitin chain probes—for example, di‐ubiquitins linked via a defined Lys can be synthesized with a warhead at the linkage to trap deubiquitinases that cleave that specific chain type (see below).

### 3.4 A Case Study—DiSUMO‐2 Vinyl Amide ABP

**FIGURE 4:**
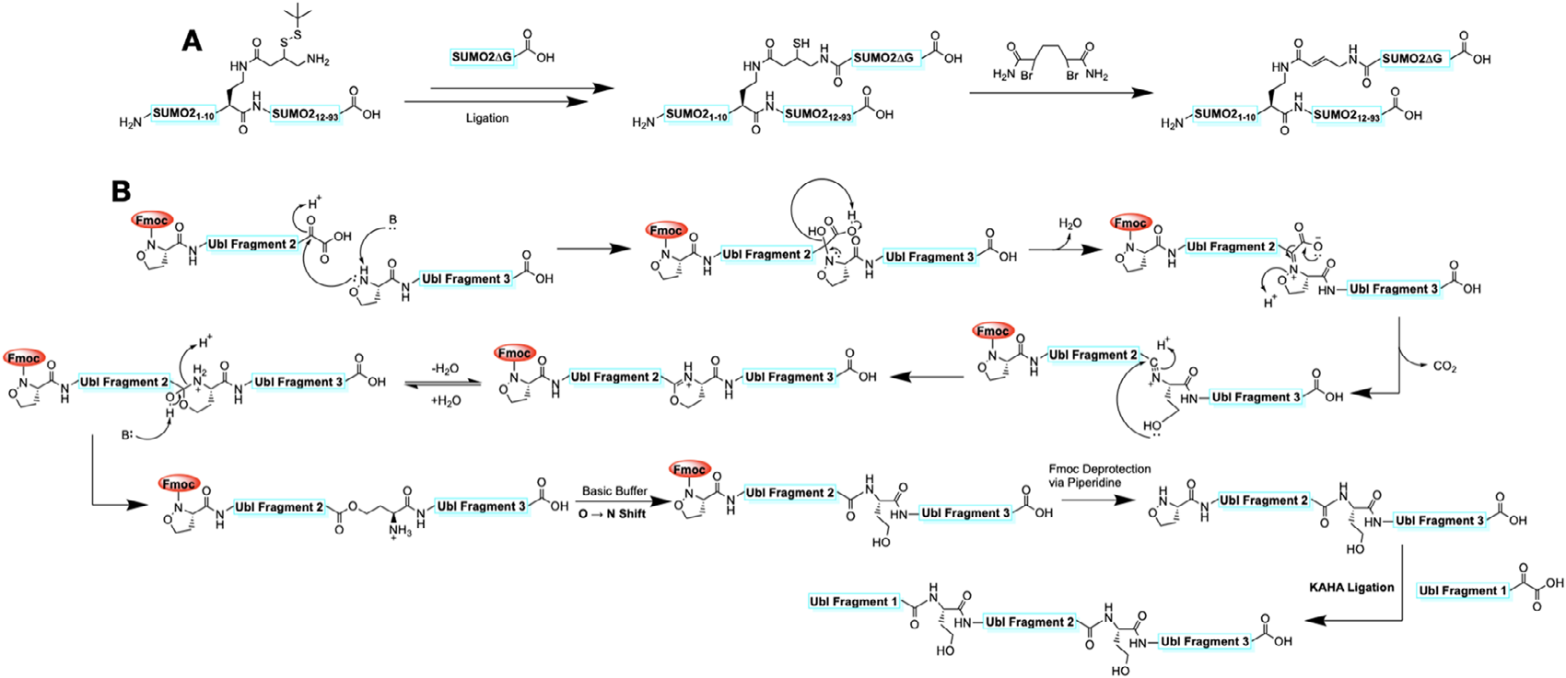
Further applications of native chemical ligation to synthesize Ubls and bioconjugate mimics. (A) The linkage of two SUMO2 proteins through the usage of a disulfide‐based thiol warhead at the Lys11 position. A thioester on a SUMO2 fragment without a glycine at the *C*‐terminus facilitates this reaction by allowing for a nucleophilic attack. (B) The KAHA ligation of a complex Ubl proceeds through multiple steps. The usage of an FMOC group to protect the 5‐oxaproline until required permits sequential addition until the correct, full‐length Ubl is formed from the precursors.

A sophisticated example of auxiliary‐mediated ligation is the diSUMO‐2 K11‐linked probe with a vinyl amide warhead reported by the Ovaa group [47]. In this probe, two SUMO‐2 molecules are linked through a warhead at the Lys11–Gly93 isopeptide junction. The synthetic scheme involved linear Fmoc SPPS of two peptide segments: (1) a SUMO‐2 (1–93) fragment where Lys11 was replaced by a diaminobutyric acid (Dab) and outfitted with a cleavable disulfide‐based thiol handle on that Dab side chain, and (2) a SUMO‐2 (12–∼92) fragment ending in a thioester. By ligating these, a precursor diSUMO with a functionalized Dab at the linkage was obtained. Then, the functionalized Dab sidechain was converted into a vinyl amide by thiol elimination. This conversion was achieved by treatment with 2,5‐dibromohexanediamide to eliminate the thiol handle, yielding a double bond between the two SUMOs. The final diSUMO‐2 probe thus contained a vinyl amide at the former isopeptide bond, an electrophile that covalently traps the active‐site cysteine of SUMO proteases (SENPs) upon binding. This probe was reactive toward all SUMO‐deconjugating enzymes in cell lysates except the NEDD8‐specific SENP8, and it revealed isoform preferences of certain SENPs. Figure 4A depicts this reaction, which illustrates how NCL plus clever auxiliary chemistry can place warheads at otherwise inaccessible internal junctions, creating chain‐mimicking ABPs for linkage‐specific proteases.

### 3.5 α‐Ketoacid‐Hydroxylamine (KAHA) Ligation

An alternative to the standard chemical ligation is Ketoacid‐Hydroxylamine, or KAHA ligation. Developed by Bode and coworkers, this technique employs the use of a full amide‐bond‐forming ligation that is not only chemoselective but also extremely clean, able to proceed in a variety of solvents, and producing only water and carbon dioxide as byproducts [51]. At the *C*‐terminus of a protein fragment is an α‐ketoacid, which reacts with an alkyl hydroxylamine found at the *N*‐terminus of another protein fragment. This subsequent reaction proceeds and releases carbon dioxide and water byproducts, and allows for the synthesis of proteins using two unprotected fragments. Bode and his group have subsequently exploited this technique to perform sequential KAHA ligation, where the *N*‐terminus of a peptide fragment has a 5‐oxaproline in the place of a serine [52]. If necessary, there will also be a protecting group (e.g., Fmoc) present on the oxaproline, which can then be removed to facilitate its own ligation to another fragment, allowing for the synthesis of longer proteins that otherwise would be impossible to reach through full SPPS methods. The mechanism for the formation of the final product has been debated, as Bode proposes several potential paths through the reaction mechanism, eventually settling on the formation of a depsipeptide before a subsequent rearrangement into the desired amide [52, 53, 54, 55]. Figure 4B shows the usage of KAHA ligation in the synthesis of a complex Ubl that would otherwise be impossible to synthesize through SPPS. Through this technique, synthetic UFM1 proteins have been synthesized by Bode with a variety of *C*‐termini.

### 3.6 Ligation Conditions and Purification

NCL is typically carried out in denaturing buffers (6 M guanidine) at pH 7‐8 with thiol catalysts like MPAA (mercaptophenylacetic acid) or TCEP to accelerate the reaction [56, 57]. After ligation, the product is folded by dialysis into a refolding buffer if necessary (for larger proteins) or directly purified by HPLC. Desulfurization (to convert Cys to Ala or remove auxiliary thiols) is done with radical initiators (e.g., VA‐044) and TCEP in the presence of t‐BuSH or other thiols [58, 59]. The final ABP product, often with > 95% purity after HPLC, can be lyophilized and stored. While total chemical synthesis is labor‐intensive, it provides complete control over the protein structure—any site can be modified (e.g., unnatural amino acids, PTMs, isotopic labels). Thus, NCL is indispensable for creating Ubl analogues that cannot be produced recombinantly. In practice, high‐end probes like the diSUMO photoaffinity probes (see below) have been assembled from multiple synthetic segments by serial ligations. The drawback is that not all labs have the capacity for such complex syntheses; nevertheless, the advancement of ligation methodology and the availability of commercial peptide synthesis services are making these approaches more accessible.

## 4 Activated Cysteine‐Directed Protein Ligation (ACPL)—Intein‐Free Semisynthesis

A recent addition to the Ub/Ubl probe synthesis toolbox is activated cysteine‐directed protein ligation (ACPL) [60]. This technique enables the generation of *C*‐terminally modified Ubls without an intein, by chemically activating a *C*‐terminal cysteine residue on a recombinant Ubl so that it undergoes acyl substitution by an incoming amine. In essence, ACPL achieves a similar outcome to intein‐EPL (a protein thioester plus aminolysis) but uses a small‐molecule reagent to activate the protein's own cysteine in situ. Key points about ACPL include:

### 4.1 Concept and Mechanism

**FIGURE 5:**
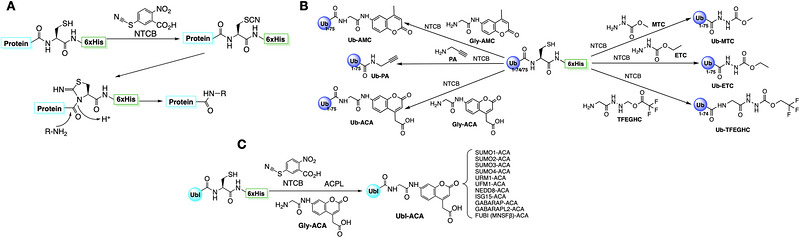
Activated cysteine‐based protein ligation (ACPL) technique and its use in the synthesis of Ub/Ubl activity and fluorogenic probes. (A) Chemical mechanism of ACPL. A protein cysteine is activated by a nitrile donor molecule, e.g., 2‐nitro‐5‐thiocyanatobenzoic acid (NTCB) to form a 1‐acyl‐2‐iminothiazolidine intermediate that undergoes nucleophilic acyl substitution with an amine to generate a *C*‐terminally functionalized protein. (B) Use of ACPL to generate ubiquitin activity‐based probes like Ub Azapeptide Esters, Ub‐PA, and newly developed fluorogenic probes such as Ub‐ACA. (C) Summary of engineered Ubls (SUMO1–4, URM1, UFM1, NEDD8, ISG15, GABARAP, GABARAPL2, and FUBI (MNSFβ) used to synthesize desired Ubl‐ACA fluorogenic probes via the ACPL method.

In ACPL, one expresses the Ubl of interest with an added *C*‐terminal cysteine (e.g., Ub_1–75_ with a Cys at position 76). This folded protein is then treated with a cyanylating agent, typically 2‐nitro‐5‐thiocyanatobenzoic acid (NTCB), which selectively reacts with the cysteine thiolate, attaching a thiocyanate group and generating an activated cysteine intermediate. The cysteine's backbone amide participates in forming a 1‐acyl‐2‐iminothiazolidine ring (a cyclic thioimidate), which is a high‐energy species [60, 61]. In the presence of a nucleophilic amine, this intermediate undergoes nucleophilic acyl substitution: the amine attacks the Ubl's carbonyl (adjacent to the cysteine), displacing the cysteine‐thiocyanate as a leaving group and forming a new amide bond. Effectively, the cysteine is replaced by the incoming amine—thus attaching a new group at the former *C*‐terminus (Figure 5A). If no external amine is provided (e.g., just buffer), the activated cysteine instead hydrolyzes, yielding a Ubl with its Cys replaced by a glycine hydrazoic acid (an oxazolone that hydrolyzes to a glycine, similar to what happens in standard NTCB “chemical cleavage”). Notably, if hydrazine is the nucleophile, ACPL produces a Ubl–hydrazide directly in one step—a convenient route to Ubl hydrazides without inteins. Overall, ACPL can be seen as a chemical mimic of the intein's “ligation” step, but using an activated cysteine in place of a thioester.

### 4.2 Application to Ubl Probes

Our previous works demonstrated ACPL's versatility by synthesizing a variety of ubiquitin and Ubl derivatives. For example, starting from Ub‐G76C (ubiquitin with a *C*‐terminal Cys), they treated it with NTCB and then added propargylamine [62]. This yielded ubiquitin‐propargylamine (Ub‐PA) in a single reaction without any enzyme. In a recent advancement of synthesizing Ub activity‐based probes, we synthesized a panel of Ub azapeptide ester probes (Ub‐MTC, Ub‐ETC, Ub‐TFEGHC) using ACPL and demonstrated that these probes covalently label their target DUBs in cell lysates and mouse tissues better than conventional probes like Ub‐PA [62] (Figure 5B). Similarly, attaching glycine‐7‐amino‐4‐coumarin (glycine‐AMC, a fluorophore for DUB assays) to ubiquitin by ACPL, Ub–AMC can be produced in one step. However, to overcome the poor solubility and high availability cost of Ub‐AMC, we recently showed one‐pot synthesis of Ub‐ACA and 12 different Ubl‐ACA fluorogenic substrates (including SUMO1‐4, NEDD8, ISG15, UFM1, URM1, GABARAP, GABARAPL2, and FUBI) by simply mixing each Ubl with a *C*‐terminal Cys with NTCB and the Gly‐ACA (Figure 5C) [63]. Five of those (e.g., UFM1‐ACA, URM1‐ACA, GABARAP‐ACA, GABARAPL2‐ACA, FUBI‐ACA) were the first ever fluorogenic substrates for those respective Ubl pathways. In essence, ACPL can produce the same probes as intein methods but often more quickly and in higher yield, since it avoids multistep purification.

### 4.3 Advantages

ACPL bypasses the need for an intein fusion and its associated drawbacks (e.g., premature cleavage, low yields under denaturing conditions). It works on standard recombinant proteins and is compatible with denatured or native conditions. In fact, one can perform the cyanylation reaction under denaturing conditions (6 M guanidine) if needed to ensure complete reaction of the cysteine. ACPL is also quick and scalable: it is essentially one chemical step after protein expression. Our previous works highlighted that ACPL is “exceedingly simple and highly useful,” vastly expanding the synthetic capacity of protein chemistry. Because it relies on a small‐molecule reagent (NTCB) and common lab chemicals, it can be implemented without special enzymes or resins. The method has been applied not only to Ubls but also to larger proteins—e.g., they modified a histone H2A (14 kDa) at its C‐terminus with an acetyl‐lysine by ACPL, and even an enzyme (RNase H) in a segment coupling strategy [60]. For Ubl ABPs, ACPL offers a fast route to warhead attachment (P, VS, etc.) or to install handles like AMC on any Ubl that can be expressed with a *C*‐terminal Gly→Cys mutation.

### 4.4 Considerations

ACPL does require that the Ubl tolerate a *C*‐terminal Cys mutation. Most Ubls naturally end in Gly‐Gly, so adding a Cys after that is usually fine (it might slightly alter recognition of some enzymes, but generally the glycine motif is the key part). In some cases, one might remove the terminal Gly (ΔGG mutant) and replace it with Cys, which can be a trade‐off in recognition versus ease of modification. If the Ubl itself has internal cysteines, those must be mutation‐neutralized or protected, since NTCB would react with them too. For example, ISG15, SUMO1‐4, and FUBI have an internal cysteine; in their ACA probe synthesis, the Cys was mutated to Ala to avoid side reactions. The chemistry of ACPL results in a leaving group and sometimes a minor side product. However, these are easily separated by desalting or nickel repurification (since the product still has a His‐tag).

In summary, ACPL is a powerful new semisynthetic route for Ubl probes. It uses a chemical activation of a terminal cysteine to achieve what inteins traditionally did—creation of a reactive *C*‐terminus—enabling direct attachment of warheads or tags in one step. ACPL has been validated on ubiquitin, SUMOs, NEDD8, ISG15, UFM1, and others. Given its simplicity and scalability (dozens of milligrams of probe can be made easily), ACPL is likely to become a standard technique alongside intein/EPL for preparing Ubl ABPs and other protein conjugates. It effectively complements the hydrazide/anhydride approach with ACPL, leveraging a cysteine handle and an amine, whereas Bode's method leverages a hydrazide handle and an anhydride. A practitioner can choose based on convenience: ACPL if expressing a Cys‐mutant is easy, or the hydrazide route if an intein fusion is already in hand.

## 5 *C*‐Terminal Hydrazide Handle and Acylation Chemistry

Peptide *C*‐terminal hydrazides have emerged as versatile intermediates in protein semisynthesis. A *C*‐terminal hydrazide can be thought of as a protected thioester equivalent: it can be converted to many other functional groups, including thioesters, via mild chemistry. Several methods exist to generate Ubl *C*‐terminal hydrazides:

### 5.1 Direct Hydrazinolysis of Thioesters or Cys‐terminated Proteins

**FIGURE 6:**
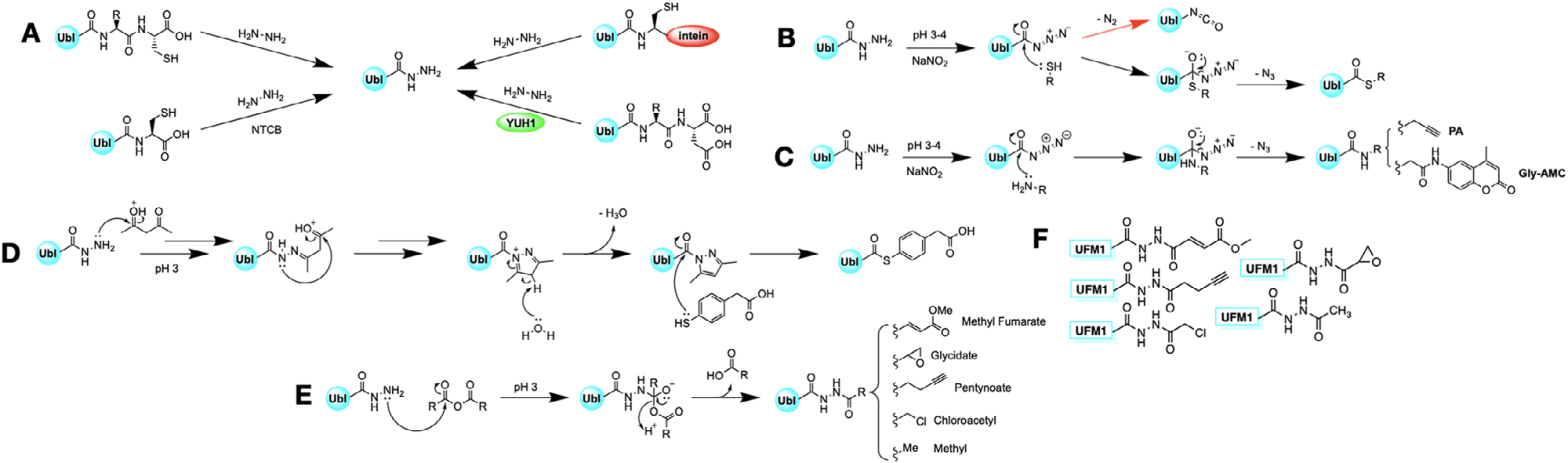
Synthesis of *C*‐terminal hydrazides on Ubls and their subsequent use in warhead installation. (A) The usage of, in counterclockwise order from the top left: N‐>S acyl shift, cyanylation of the *C*‐terminus, cleavage of an Asp mutant with Yuh1, and intein‐mediated hydrazine synthesis. (B) Activation of the hydrazide handle by sodium nitrite and the subsequent paths of either Curtius rearrangement (marked in red), or the desired attack by a thiol to yield a thioester. (C) The activation of the hydrazide handle was followed by an attack by an amine and several previously synthesized probes. (D) Acetylacetone in Knorr Pyrazole Synthesis allows subsequent attack by mercaptophenylacetic acid. This undergoes a similar activation that allows for an attack with an amine to generate probes. (E) Anhydride acylation of a *C*‐terminal hydrazide and warheads that have been attached through this method. (F) Ubiquitin Fold Modifier‐1 (UFM1) probes that were synthesized using the anhydride acylation method.

One route is to express the Ubl with an extra *C*‐terminal cysteine (e.g., Ub(1–76)‐Cys) and treat it with hydrazine under conditions at 50 °C that induce an N→S acyl shift at the Gly76‐Cys bond. This leads to cleavage of the Gly–Cys bond and the formation of Ubl hydrazide. MacMillan and coworkers showed that ubiquitin with a C‐terminal Cys can undergo such an N→S acyl transfer in neat hydrazine, yielding Ub–hydrazide in good yield (∼50 mg/L culture) [64]. Another clever enzymatic approach is to utilize the ubiquitin‐specific protease Yuh1: a ubiquitin mutant with an extra Asp (Ub(1‐76)‐Asp) was cleaved by Yuh1 in the presence of hydrazine, trapping the *C*‐terminus as a hydrazide (this works because the enzyme forms an acyl‐enzyme intermediate that is intercepted by hydrazine) [65, 66]. This yielded even higher amounts of Ub hydrazide (∼30–40 mg/L). ACPL method uses cyanylating reagents (e.g., 2‐nitro‐5‐thiocyanatobenzoic acid, NTCB) to activate the *C*‐terminus for hydrazinolysis—treating a recombinant protein cysteine with NTCB and hydrazine directly installs a *C*‐terminal hydrazide [60, 67]. All of these techniques lead to formation of a hydrazide, which can then be leveraged for further acylation (Figure 6A).

### 5.2 Intein‐Mediated Hydrazides

As a variant of the intein method discussed above, instead of thiol cleavage, one can cleave the intein fusion with hydrazine. An intein with a suitable mutation will allow transamidation by hydrazine, releasing the protein as a *C*‐terminal hydrazide conjugate [68]. This approach similarly affords high yields and has been applied to several Ubls. For example, UFM1‐hydrazide was prepared recombinantly by intein cleavage into hydrazine. Because hydrazine can be used at high concentrations without denaturing the protein severely (especially at low temperature), the released Ubl‐hydrazide is often folded.

Once a Ubl *C*‐terminal hydrazide is in hand, it can be chemically converted to multiple other forms, enabling flexible synthesis of probes:

### 5.3 Conversion to Thioesters

Ubl hydrazides can be transformed into thioesters by a two‐step “activation and thiolysis.” The classic route is treating the hydrazide moiety with sodium nitrite (NaNO_2_) under acidic conditions (pH ∼3–4) to form a *C*‐terminal acyl azide. The acyl azide can either undergo Curtius rearrangement or, more usefully, be attacked by a desired nucleophile (Figure 6B). If a thiolate salt like MESNa is added before the acyl azide decays, one obtains the corresponding thioester [69]. For instance, UFM1 hydrazide was oxidized with NaNO_2_ to the acyl azide and then reacted with MESNa, yielding a UFM1‐MESNa thioester, (Alternatively, one can add a cysteine or other thiol‐containing peptide to form a ligation product directly via hydrazide‐based NCL, a common technique in protein total synthesis known as KCL (Kennel Chemical Ligation).

### 5.4 Direct Acylation With Electrophiles (Warhead Installation)

The *C*‐terminal acyl azide can also be intercepted by amines to form amide‐linked warheads (Figure 6C). For example, after NaNO_2_ activation of UFM1 hydrazide, addition of propargylamine produced UFM1‐propargylamine (UFM1‐PA) in one step [70]. Similarly, reaction with glycine‐7‐amino‐4‐methylcoumarin yields a fluorogenic Ubl‐AMC probe. In general, this “acyl azide capture” method is a convenient way to attach small molecule warheads that have a single nucleophilic site (amine, hydrazide, etc.) to the Ubl *C*‐terminus.

### 5.5 Knorr Pyrazole Synthesis Activation

Another method of activation of the hydrazide is through the use of acetyl acetone. The hydrazide handle attacks the carbonyl as a hydrazine would under acidic conditions. The subsequent hydrazone intermediate is then turned into a pyrazole, which then can be attacked by an aryl thiol (4‐Mercaptophenylacetic acid, or MPAA in this case) to create another thioester as depicted in Figure 6D. This aryl thioester is suitable for either native chemical ligation or the synthesis of an aryl electrophilic probe, which is permitted by the aryl nature of the pyrazole [71].

### 5.6 One‐Step Anhydride Acylation (Bode's Method)

A recently developed, extremely convenient method avoids even the NaNO_2_ activation. Bode and coworkers demonstrated that at pH ∼3, the α‐amine groups of a folded protein are protonated and unreactive, whereas the terminal hydrazide can be selectively acylated by an acid anhydride (Figure 6E) [72]. Thus, simply mixing Ubl hydrazide with a symmetric anhydride of the desired warhead precursor leads to site‐specific acylation on the hydrazide nitrogen. This yields a Ubl acyl hydrazide adduct carrying the warhead moiety. Crucially, the protein stays folded, and other side chains (Lys, etc.) remain unmodified at low pH. Using this strategy, various electrophilic groups have been attached in one step: methyl fumarate (maleimide‐like double bond), glycidate (epoxide), 4‐pentynoate (terminal alkyne), and even chloroacetyl (haloacetyl) warheads were all installed on UFM1 hydrazide by treatment with the corresponding anhydride. The result is, for example, UFM1–CO–NH–NH–CO–CH = CH–COOCH_3_ (a methyl fumarate adduct) or UFM1–CO–NH–NH–CO–CH_2_Cl (a chloroacetyl adduct). Despite the extra acyl linkage (the –NH–NH–CO– bridge), these adducts function as ABPs: the warhead can react covalently with a target cysteine, tethering the Ubl to the enzyme. In fact, a chloroacetyl‐modified UFM1 probe displayed remarkable selectivity for the UFSP2 protease in cell lysates and live cells, labeling virtually only the target enzyme. Nonspecific reactivity was minimal, highlighting the clean reactivity of the haloacetyl warhead under physiological conditions. The beauty of Bode's method is that it “democratizes” ABP synthesis, enabling a biochemistry lab to take a recombinant Ubl‐hydrazide and, by adding a single reagent, obtain a ready‐to‐use probe without chromatography. This was further shown by generating NEDD8 (ΔGG) and SUMO‐2 (ΔGG) probes with various electrophiles directly from hydrazides, avoiding multi‐step preparations. Those probes retained specificity (e.g., a SUMO‐2 probe acylated with an electrophile selectively labeled SENP1 in assays) [66].

After acylation, excess anhydride or reagents are simply quenched or dialyzed away, and the probe can be used directly or briefly purified. The mild conditions preserve the tertiary structure—studies confirmed that acyl‐hydrazide Ubl probes are folded and recognize their target enzymes properly. In summary, *C*‐terminal hydrazide chemistry provides a highly flexible platform: from one Ubl‐hydrazide, one can generate a panel of ABPs by choosing different electrophile reagents (e.g., VS, epoxide, alkyne, etc.) as shown in Figure 6F. This “warhead diversification” is extremely useful in probing different enzyme classes or optimizing reactivity. Notably, certain warheads like acyloxymethyl ketones and haloalkanes that are unstable to long syntheses can be introduced last‐minute via hydrazide acylation, ensuring the final product is obtained in high yield and purity.

## 6 Full Solid‐Phase Peptide Synthesis (SPPS) Routes for Ubls and Variants

**FIGURE 7:**
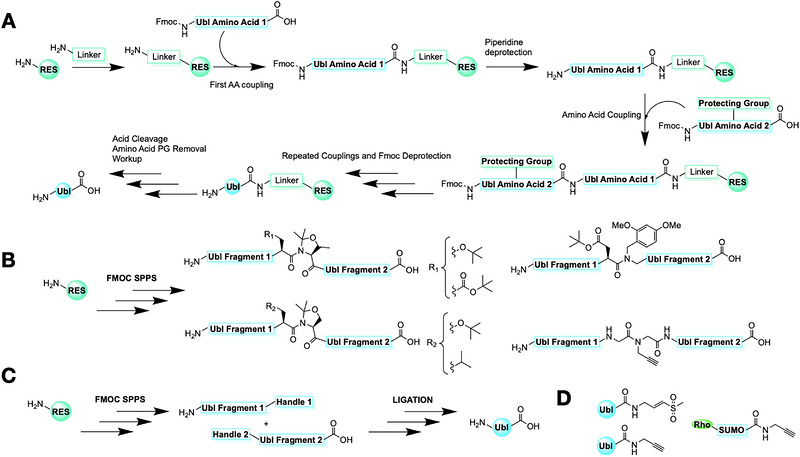
Applications of Solid Phase Peptide Synthesis in Ubls and their ABPs. (A) A general mechanism for the solid‐phase peptide synthesis of Ubls. (B) The usage of “aggregation breakers” in order to ensure proper folding of the protein and prevent aggregation while on the resin. (C) The synthesis of separate fragments of a Ubl via SPPS and their subsequent ligation by using handles present. (D) Potentialally synthesizable Ubls along with a Rhodamine‐SUMO‐PA ABP, synthesized by Ovaa et al.

Despite their size, many Ubls (∼75–100 amino acids) have been successfully produced by total chemical synthesis, which allows unparalleled freedom to introduce modifications. Fmoc‐based SPPS (as depicted in Figure 7A) can typically assemble peptides up to ∼50 residues; to obtain full‐length Ubls, chemists use either segmented synthesis (with NCL, as described) or advanced SPPS techniques to push the length limits. Here, we outline the strategies for synthesizing Ubls and ABP analogs entirely chemically:

### 6.1 Direct SPPS of Full‐Length Ubls

With optimized protocols, researchers have made Ubls in one piece on resin (Figure 7B). Key to success are “aggregation breakers”—special dipeptide motifs (like pseudoproline dipeptides or dipeptidyl diisopropylamides) inserted at intervals to disrupt secondary structure formation during elongation. Ovaa et al. employed such tactics to synthesize a 76‐residue ubiquitin thioester and even a 92‐residue SUMO‐2 via SPPS [47, 73]. In those cases, the use of pseudoproline at selected positions and rigorous coupling protocols (HATU or PyBOP activators, double couplings, etc.) yielded full‐length chains with manageable purities. After chain assembly and cleavage from the resin (typically with TFA cocktails), the crude peptide is purified by reverse‐phase HPLC [74]. If the peptide is a thioester (requiring specialized resin or Dawson's “safety‐catch” linkers), it can be directly used in ligation; if it's a full‐length Ubl, it may require folding in vitro. In Ovaa's report, synthetic SUMO‐2 folded into its native β‐grasp tertiary structure and was functional, underscoring that total synthesis is feasible for Ubls given modern peptide chemistry. Brik and coworkers have also employed a similar method using propargylation to act as a secondary structure disruptor as well, removing this group through the use of Gold(I) Chloride [75].

### 6.2 Fragment Synthesis and Ligation

As discussed, a more common approach is to break the Ubl into two or more fragments that are individually synthesized and then ligated (Figure 7C). For example, Brik's two‐fragment Ub synthesis, or other multi‐fragment routes for larger proteins, rely on robust SPPS of each fragment (usually < 40–50 residues each for reliability) [46]. Each fragment may incorporate unique functional handles: one fragment might be a thioester (using an appropriate linker or the sulfurization method on an Ala, for instance), and the other fragment starts with an N‐terminal Cys (or Ser/Thr that can be converted to Cys via thiolysis of an *N*‐terminal thiazolidine). Fragments are purified and ligated in sequence. After each ligation, one might remove a cysteine (if non‐native) by desulfurization. For very large constructs or branched structures, iterative ligation can be done (even on solid support or in one‐pot sequentially). The isopeptide‐linked diSUMO‐2 mentioned earlier was actually assembled from four peptide segments (since two SUMO units were made, each from two pieces) using hydrazide‐based ligation methods. Such complex synthetic schemes are challenging but demonstrate the power of chemical synthesis in accessing protein architectures beyond the reach of genetic methods.

### 6.3 Incorporation of Unnatural Amino Acids and Warheads

Full SPPS provides the maximal flexibility for installing nonstandard functionalities (Figure 7D). One can introduce unnatural amino acids at any position by using modified amino acid building blocks. For example, p‐benzoylphenylalanine (Bpa) can be directly coupled where a photo‐crosslinker is needed, or a diazirine‐bearing amino acid can be included for photoreactive probes [76, 77]. Dehydroalanine (Dha) can be introduced by SPPS in multiple ways: a direct approach is to use an amino acid like Fmoc‐Ser(O‐t‐Bu)‐t‐Bu (for example) and then eliminate to Dha post‐synthesis, but a more common approach is to synthesize a cysteine‐containing peptide and convert Cys to Dha on the resin or in solution [78]. In the UFM1‐Dha probe example, the peptide was synthesized with a *C*‐terminal Cys(benzyl) ester; after assembly, an oxidative elimination with O‐mesitylenesulfonylhydroxylamine (MSH) was performed to yield a dehydroalanine at the *C*‐terminus [79]. This illustrates that unique warhead precursors can be built into the peptide and then revealed by tailored chemical reactions. Another common tactic is to incorporate a Sec (selenocysteine) or cysteine at a position and then alkylate or eliminate it to form a warhead or mimetic (selenocysteine can be deselenized to alanine or used in ligation to enable ligation at normally inaccessible junctions) [80, 81].

Electrophilic warhead moieties can sometimes be introduced as modified amino acids. For example, to get a vinyl sulfone at the *C*‐terminus purely by SPPS, one could couple a pre‐formed vinyl sulfone acid as the last residue. Or to make a propargylamine via SPPS, one might incorporate a Gly that is pre‐coupled to propargylamine as a handle. In practice, it is often easier to synthesize the fully deprotected Ubl and then do the final warhead attachment by solution‐phase chemistry (as described in the hydrazide section or via activated esters).

### 6.4 Fluorophores or Tags Can be Attached on‐resin

For example, 5(6)‐TAMRA (carboxytetramethylrhodamine) can be introduced by coupling its NHS ester to an *N*‐terminal aminooxyacetyl group, or more directly, by using commercially available Fmoc‐Lys(ivDde)‐biotin or Fmoc‐Lys(Mmt)‐TAMRA building blocks. In the SUMO‐PA probe work by Ovaa's team, they simply incorporated an N^α–Boc protected 5‐carboxyrhodamine (Rhodamine110) dye during SPPS at the *N*‐terminus (Figure 7D) [82]. After completing chain assembly (including the propargyl warhead at the *C*‐terminus), cleavage provided a SUMO probe already bearing a fluorescent dye for imaging.

### 6.5 Folding and Verification

Chemically synthesized Ubls often require folding by dilution and redox shuffling if they contain disulfide bonds (most ubiquitin‐like proteins do not have disulfides, except SUMO has none either) [82]. Ubls typically refold spontaneously when the peptide is of high purity, as the native sequence is inherently stable. For ubiquitin, which has no cysteines in its sequence, folding is usually not an issue; for others like ISG15 (two‐domain Ubl) or FAT10 (two‐domain), the synthetic approach might involve folding each domain and then ligating them, or careful refolding of the full sequence.

### 6.6 Advantages Versus challenges

Full SPPS and total synthesis allow incorporation of multiple novel elements in one probe—for example, a SUMO‐2 probe was made with both a propargylamine warhead and a photocaging group by chemical synthesis (discussed below). This level of multi‐functionalization is hard to achieve in any other way. The main challenge is that it requires expertise in peptide chemistry and access to HPLC, etc. Yields of full‐length product can be modest (milligram scale), but that is usually sufficient for biochemical experiments. The expense can be nontrivial if outsourced (dozens of amino acids with modifications). Because of this, commercial suppliers generally do not offer full‐length Ubls with complex warheads aside from a few popular ones—it's more efficient for them to produce those via semisynthesis. As of 2025, a few specialized companies sell synthetic ubiquitin variants or peptides (e.g., UbiQ and LifeSensors sell ubiquitin with certain modifications like alkyne handles or phosphorylations), but for most custom ABPs, in‐house synthesis or academic collaborations are the way to obtain them. Nevertheless, the field has seen rapid progress, and synthetic Ubl probes are becoming more routine as methodologies like automated ligation and hydrazide strategies simplify the workflow.

## 7 Enzymatic Approaches

### 7.1 Sortase‐A Mediated Ligation

**FIGURE 8:**
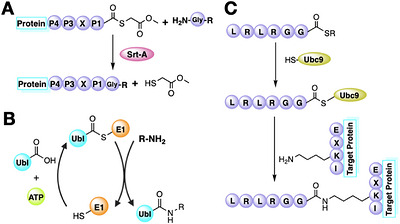
Enzymatic routes to Ubl activity‐based probes. (A) Sortase installs electrophiles at the engineered P4‐P3‐X‐P1 terminus, thioester‐mediated sortagging drives ligation irreversibly. (B) ATP‐driven E1 enzyme‐mediated thioester activation initiates Ubl conjugation by forming a high‐energy E1–Ubl thioester intermediate. These can be intercepted by exogenous nucleophiles (PA, VS, etc.) to generate Ubl probes. (C) The LACE method uses SUMO E2 (Ubc9) to transfer Ubls onto lysines within IKXE motifs. Covalent E2–Ubl conjugates equipped with electrophilic linkers trap E1s and E3s via active‐site cysteines.

Sortase‐A (SrtA) catalyzes transpeptidation between an *LPXTG* motif and an N‐terminal Gly_n_ nucleophile [83, 84]. In Ubl probe synthesis, Ubl–LPETG constructs can be ligated to Gly_3–_warhead or Gly_3–_tag peptides, installing electrophiles or reporters under mild aqueous conditions (pH 7–8, Ca^2^^+^‐dependent). Thioester‐assisted sortagging replaces the terminal Gly with a thioester donor, driving the ligation irreversibly and expanding sequence tolerance (Figure 8A) [85].

### 7.2 E1 Mediated Thioester Activation and Transfer

Ubl E1 enzymes activate Ubls via ATP‐dependent adenylation, forming thioesters with their catalytic cysteine (Figure 8B) [86]. These intermediates can be diverted to different small molecule amines producing Ubl‐VS, Ubl‐VME, and Ubl‐PA probes. Dehydroalanine (Dha)‐modified Ubls act as “cascade probes,” covalently labeling E1, E2, and E3 cysteine enzymes upon activation [26]. E1‐mediated strategies ensure correct *C*‐terminal activation, yield high‐fidelity folded products, and allow selective targeting of conjugation enzymes across all Ubl families (Ub, SUMO, NEDD8, UFM1, ATG8/12). Limitations include enzyme specificity and the need for optimized nucleophile trapping.

### 7.3 E2/E3‐Based Transligation and Ligation Strategies

E2 enzymes (notably SUMO E2 Ubc9) catalyze direct substrate modification. As shown in Figure 8C, the LACE (Lysine Acylation using Conjugating Enzymes) method exploits Ubc9's recognition of the IKXE motif to transfer Ub, SUMO, or ISG15 onto tagged lysines using Ubl–thioesters [87]. An engineered chimeric E1/E2 pair further enhances yield and enables the co‐expression of monoubiquitinated proteins [88]. HECT and RBR E3s transiently form thioesters with Ubls, making them direct ABP targets. Covalent E2–Ub conjugates with electrophilic linkers (vinyl sulfone, acrylamide) capture E1s and E3s (e.g., Parkin, NEDD4) by trapping active‐site cysteines. Engineered E3s or substrate‐fusion constructs can generate site‐specific Ubl conjugates for ULP or protease assays.

## 8 Other Emerging Strategies

Beyond the major methods above, several innovative strategies are emerging to construct Ubl probes with special functionalities:

### 8.1 Photocaged and “On‐Demand” Activatable Probes

**FIGURE 9:**
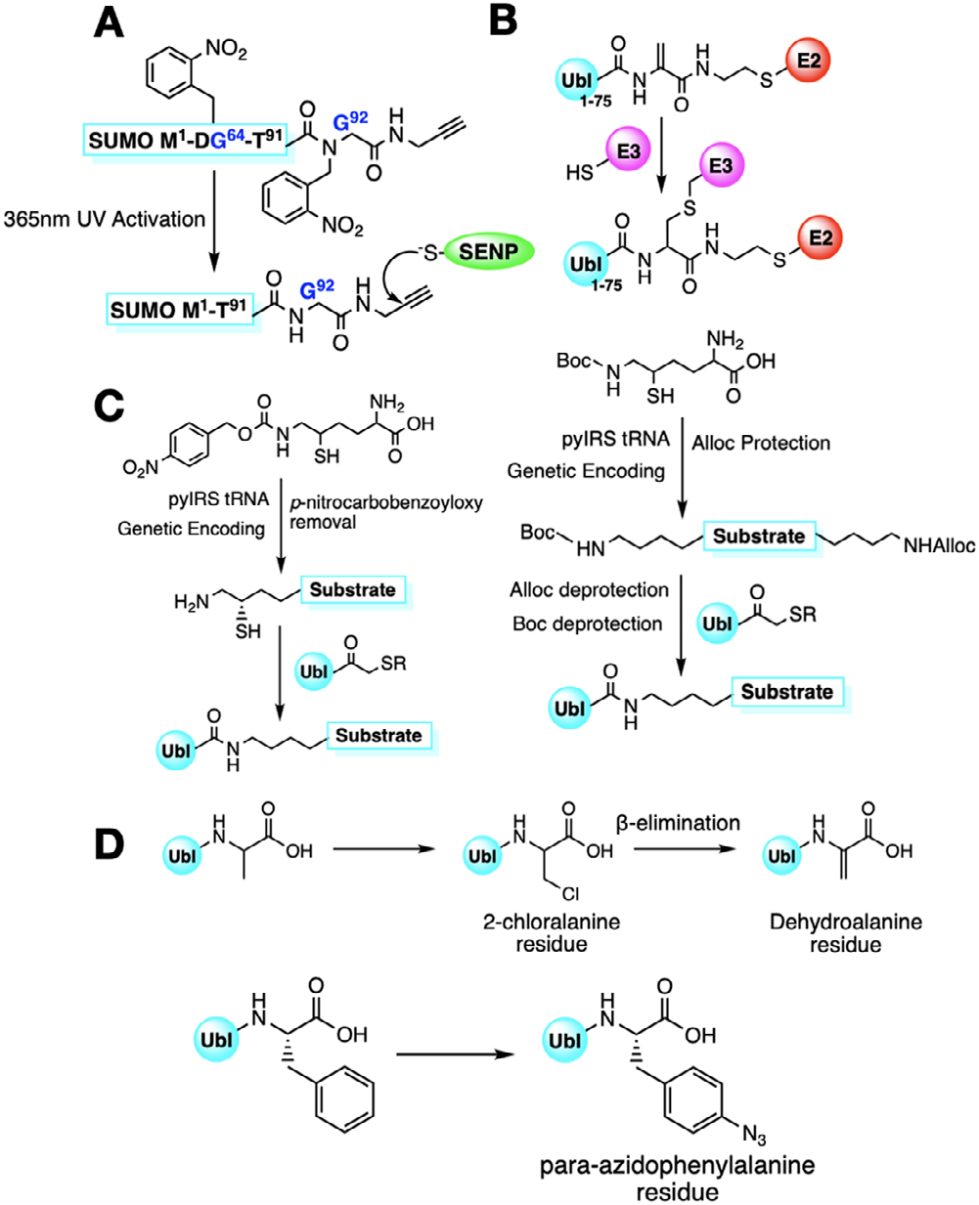
Emerging methods to synthesize different Ubl probes. (A) Photocaged Ubl probes enable precise temporal activation by incorporating photolabile protecting groups (e.g., caged glycine) at key positions. UV irradiation removes these groups, releasing the reactive warhead (e.g., PA) and allowing selective engagement with target proteases in vitro or in live cells. (B) Dual electrophile diUb or E2∼Ubl cascade probes are designed to simultaneously trap enzymes at multiple active sites (e.g., exosite and catalytic site), or across sequential enzymatic cascades. These semisynthetic constructs incorporate multiple warheads such as dehydroalanine (Dha) for sequential Michael addition and covalent capture. (C) Site‐specific Ubl ligation via orthogonal Boc and Alloc protection. A target lysine is Boc‐protected for selective Ubl conjugation, while other amines are masked with Alloc. Sequential deprotection enables precise Ubl attachment under mild conditions. (D) Genetic code expansion strategies allow direct expression of Ubls with latent warhead precursors (e.g., 2‐chloroalanine or azidophenylalanine). After translation, chemical activation (e.g., elimination to Dha or reduction to alkyne) converts the precursor into a reactive electrophile for probe installation.

One recent approach addressed the challenge of probe reactivity during synthesis and in cells by incorporating photolabile protecting groups into the Ubl (Figure 9A). Chen et al. reported a SUMO‐2 probe that was synthesized with two photocaged glycine residues—one at the *C*‐terminus (G92) to mask the propargyl warhead, and one at an internal position (G64) to prevent side reactions (aspartimide formation) during SPPS [89]. The fully caged SUMO‐2‐PA was inert (unable to react with SENP proteases) until exposed to UV light, which simultaneously removed the cages. Upon light activation, the probe rapidly engaged and covalently captured SENP proteases both in vitro and even within intact cells. This strategy offers temporal control—researchers can introduce the inert probe into cells (e.g., via microinjection or cell‐permeabilization techniques) and then activate it at a chosen time to label active enzymes, enabling time‐resolved profiling of Ubl protease activity [90]. Photocaging can be applied to other warheads or other positions (e.g., caging a critical recognition residue to inhibit binding until uncaged). Such elaborate synthetic designs underscore the level of control achievable with chemical synthesis, at the cost of additional synthetic steps for installing caging groups.

### 8.2 Dual‐Reactivity and Bifunctional Probes

Some advanced probes incorporate two electrophiles or two reactive sites to crosslink an enzyme to a substrate or to capture multi‐subunit complexes, as depicted in Figure 9B. For example, “biselectrophile” diubiquitin probes have been devised to trap a DUB simultaneously at two points (active site and an exosite) [91]. These are highly specialized and rely on multi‐step semisynthesis. Another concept is chain‐elongation probes: for example, an E2∼Ub conjugate where Ub carries a warhead so that after the E2 transfers it to an E3, the warhead locks the E3 (this is effectively a *cascading ABP*). In 2019, Xu et al. created an E2‐Ub conjugate with a *C*‐terminal dehydroalanine on ubiquitin; this probe first binds an E1, forms E2∼UbDha, then transfers to a HECT E3 where it performs Michael addition, covalently linking the ubiquitin to the E3's catalytic cysteine. This sequential trapping allowed the capture of a HECT ligase (NEDD4L) through the full E1→E2→E3 cascade, illustrating a creative way to target enzymes (like E3s) that do not directly interact with free ubiquitin. While such probes are complex (in this case requiring an expressed E2∼Ub conjugate and a chemical DHA installation), they expand the reach of ABPs beyond single‐enzyme targets to entire pathways [92].

### 8.3 Genetic Encoding and Orthogonal Amine Protection

Another method of ubiquitination by genetic encoding is through Alloc and Boc protection of amine groups that allows for site‐specific ligation of Ubls and substrates (Figure 9C) [93]. A *tert*‐butyloxycarbonyl (Boc) group is attached to a lysine, which is then bound to the substrate of interest at the site where an isopeptide linkage with the Ubl is required. The Boc‐protected substrate is then protected with allyloxycarbonyl (Alloc) at all other amine residues, providing specificity toward the amine of interest [94, 95]. The Boc group is removed by Boc deprotection, and the substrate can be treated with the Ubl, creating a Ubl‐Substrate ligation at the unprotected amine group. The ligated product then undergoes Alloc deprotection and refolding of the protein to its native structure. Alloc is a preferred protecting group over past methods like carbobenzyloxy (Cbz) protection because it is orthogonal to Boc and is removed through less harsh conditions than Cbz [96].

### 8.4 Genetic Code Expansion and Unnatural Amino Acids

Another emerging strategy is to use genetic code expansion to incorporate warhead precursors at the Ubl *C*‐terminus during expression (Figure 9D). For instance, one can engineer a stop‐codon suppression system to insert an amino acid analog with a latent electrophile (such as 2‐chloroalanine or para‐azidophenylalanine) [97]. After expression, the analog can be converted in vitro to an active warhead: for example, 2‐chloroalanine can undergo β‐elimination to yield dehydroalanine in the protein context [97]. This approach was hinted at by Mulder et al. in a report on site‐specific ubiquitination via DHA chemistry by genetically introducing a cysteine or modified residue and then chemically converting it [26]. Although not yet routine for ABPs, this approach could allow production of Ubl‐Dha or Ubl‐alkyne probes directly in cells or in bacterial expression, simplifying purification (since the protein is made with the modification built in, needing only one chemical step to activate the electrophile). Challenges include the efficiency of unnatural amino acid incorporation and the subsequent chemoselective conversion, but ongoing advances in in vivo unnatural amino acid incorporation make this a promising avenue.

### 8.5 Bis(2‐sulfanylethyl)Amino (SEA) Thioester Surrogates

**FIGURE 10:**
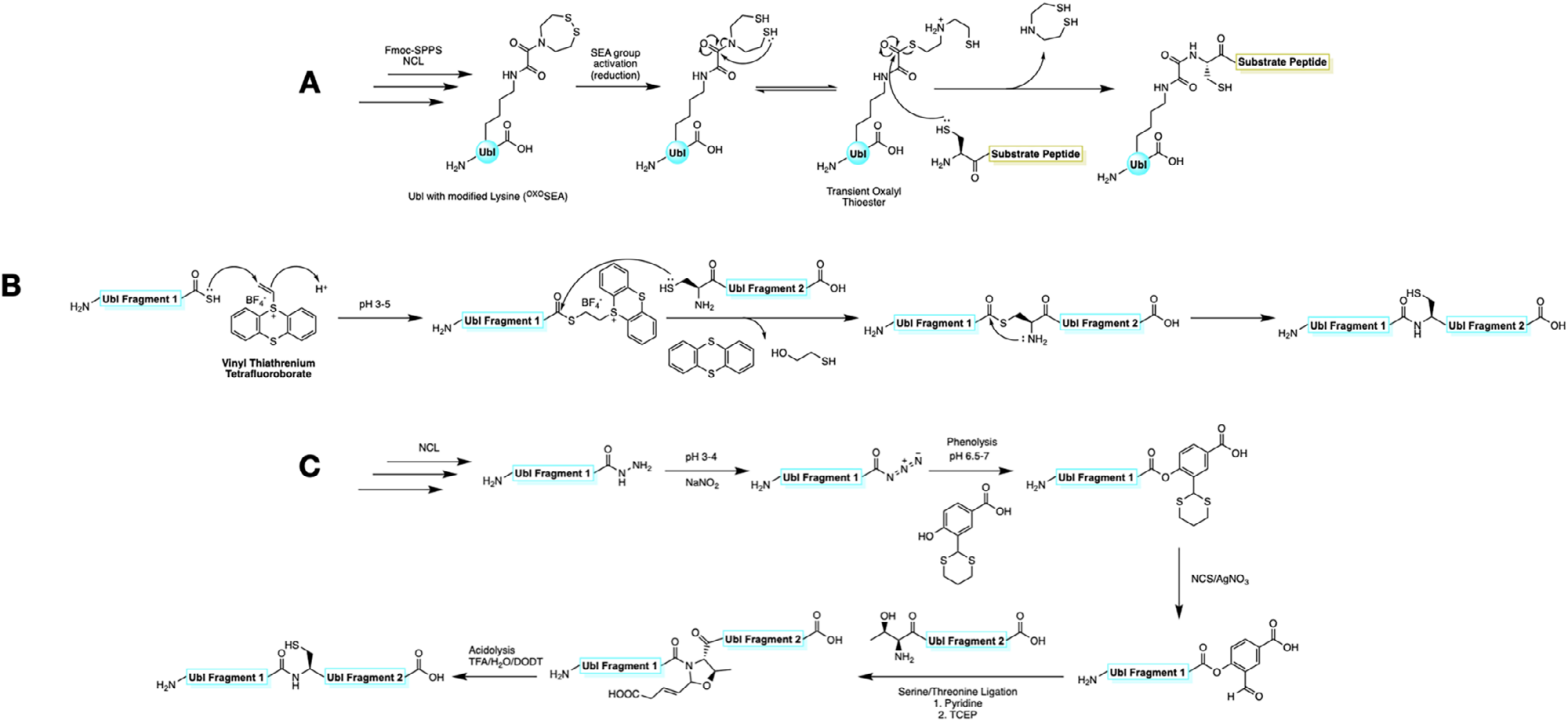
Emerging methods for the synthesis of Ubl probes and conjugates. (A) SEA thioester surrogates proposed for use in the synthesis of a Ubl conjugate. Here, after a combination of Fmoc‐SPPS and NCL, a Ubl with a modified lysine to an ^OXO^SEA group is activated through reduction. This reduction opens up the ring and allows for a rearrangement to a transient oxalyl thioester, which then can be attacked by a cysteine‐containing substrate peptide to form the conjugate. (B) The usage of Vinyl Thiatrenium Tetrafluoroborate (VTT) permits additive‐free ligation. Starting with a Ubl fragment with a terminal thioester, the VTT is attached to the Ubl fragment, making for an optimal position for attack by a secondary fragment containing a cysteine similar to a native chemical ligation. (C) The SAL ester formation is similar to the hydrazide techniques mentioned previously. Beginning with a terminal hydrazide fragment, phenolysis occurs, followed by activation into a reactive peptide SAL ester. This intermediate is primed for Serine/Threonine Ligation (STL), which can be performed with a second fragment with a threonine at the *N*‐terminus. Subsequent acidolysis leads to the formation of the final product.

One technique that is seeing recent breakthroughs is the synthesis of probes through SEA, or Bis(2‐sulfanylethyl)amino thioester surrogates (Figure 10A). Previously used in the synthesis of SUMOs 1, 2, and 3, a recent advance has shown itself in the form of oxo‐SEA, built around the foundation of the N‐oxalyl perhydro‐1,2,5‐dithiazepine handle [98, 99]. This handle is a redox‐controlled thioester precursor, which can be placed on the side chain of a lysine residue and then accessed through solid‐phase peptide synthesis. Specifically, in the context of Ubiquitin and its family of proteins, this ligation technique has allowed the use of the SEA thioester surrogates in the synthesis of SUMO‐peptide conjugates, offering a method of unique insight into identifying amino acids that help maintain a Ubl's structure. Boll and coworkers employed the technique first in 2014 to great effect in this effort, where they used native chemical ligation to first obtain a SUMO‐1 protein with an SEA group at the C terminus, which was then activated to ligate the protein with a peptide fragment to make a SUMO1‐peptide conjugate. More recently, Grain et al. have employed a more developed SEA, ^oxo^SEA, an oxalic acid‐derived thioester precursor that can be added onto the modified lysine [100]. Its reduction by an additive (e.g., TCEP) can lead to a rearrangement that allows the SEA handle to react with a peptide possessing a cysteine. Further application of this technique to the synthesis of other Ubl‐peptide conjugates and identifying their structurally significant amino acids.

### 8.6 Vinyl Thianthrenium Additive‐Free Native Chemical Ligation

As shown in Figure 10B, another technique recently developed is vinyl thianthrenium additive‐free native chemical ligation (VTANCL) [101]. A further development of the initial native chemical ligation technique, it allows for an expansion of the synthesis methodology of ABPs through the absence of additives. Using vinyl thianthrenium tetrafluoroborate, Li and coworkers have been able to synthesize proteins with this VTANCL technique. The lack of an additive allows for a reduction of cost and complexity, working in tandem with its one‐pot nature to prove to be one of the next steps forward in the development of ligation techniques. The principle of this is “selective esterification,” starting with an unprotected *C*‐terminal thioester's linkage to the vinyl thianthrenium. Subsequently, the second peptide fragment, with an *N*‐terminal cysteine, can allow for a reaction reminiscent of the native chemical ligation, with the added benefit of not requiring any additives. This technique has been used in the synthesis of native ubiquitin to great effect, and subsequent studies can focus on employing it for other Ubls.

### 8.7 Salicylaldehyde Esters

Peptide salicylaldehyde (SAL) esters represent a step forward by Lin and coworkers to (Figure 10C) facilitate protein synthesis by combining serine/threonine ligation (STL) and native chemical ligation (NCL) [102]. These SAL esters are perfect targets for STL, which is beneficial over NCL due to its chemoselectivity, allowing it to complement the previously developed technique. The method of obtaining the protein SAL ester begins with either SPPS or expression in order to obtain a protein with a C‐terminal hydrazine. Subsequently, a reaction with sodium nitrite, and then phenolysis by 3‐(1,3‐dithian‐2‐yl)‐4‐hydroxybenzoic acid (SAL(−COOH) followed by activation by either N‐cholorosuccinimide or sodium nitrate permits formation of the protein SAL ester. This ester can then be used in sequential ligation—Lin et al. have combined native chemical ligation in the formation of several initial fragments of Ubl‐5 (ubiquitin‐like protein 5), followed by the usage of STL to ligate on further amino acids and form the full protein. The technique's application in this synthesis proves its viability in the synthesis of Ubls and can and should be further employed in other Ubl syntheses.

## 9 Tagging Strategies for Detection and Enrichment of Ubl‐Based Probes

Detection and enrichment of enzyme–probe complexes are central to the utility of activity‐based probes (ABPs). Ubiquitin and ubiquitin‐like protein probes are frequently functionalized with affinity or reporter tags to enable visualization, enrichment, or tracking of labeled enzymes. Tagging strategies fall into two broad categories: (1) built‐in reporter tags, where the tag is introduced during probe synthesis or expression, and (2) bio‐orthogonal reactive handles, enabling post‐labeling conjugation of detection tags via click chemistry. Each approach offers unique advantages depending on the experimental context.

### 9.1 Built‐In Reporter Tags

*N*‐terminal tagging is the most common and structurally permissive method, as the *N*‐terminus is typically distant from the *C*‐terminal tail responsible for enzyme recognition. Alternatively, tags can be placed on solvent‐exposed loops via lysine residues or engineered cysteines. Biotin is widely used for streptavidin‐based pull‐downs and can be introduced during SPPS (e.g., Fmoc‐Lys(biotin)‐OH) or post‐synthetically via NHS/maleimide chemistry—commonly targeting *N*‐terminal cysteines [103]. Linkers like Ahx or Gly‐Ser reduce steric hindrance. Epitope tags (HA, FLAG, His_6_) facilitate immunodetection and probe purification [104]. HA–Ub–VME is a classic example; His_6_ also serves for Ni‐affinity purification. These tags are often spaced by short linkers to preserve probe function. Fluorescent dyes (e.g., TAMRA, Cy5) enable in‐gel or live‐cell imaging and are conjugated to *N*‐termini or side chains. For instance, TAMRA‐labeled SUMO‐2–PA has been used to track SENP activity [47]. Larger tags (e.g., HaloTag, SNAP‐tag) allow modular detection in two‐step workflows using reactive ligands and conjugated reporters. Though less common, they offer flexibility in probe design [105, 106].

Built‐in tags streamline probe purification (e.g., His_6_ or HA affinity purification) and simplify detection. Importantly, placement at the *N*‐terminus with suitable linkers generally preserves Ubl structure and function. However, bulky tags may hinder cell permeability or enzyme recognition in sensitive contexts.

### 9.2 Bio‐Orthogonal Handles and Click Chemistry

An increasingly popular approach is the incorporation of small, inert chemical handles (e.g., terminal alkynes or azides) into the probe structure, which can be conjugated post‐labeling using click chemistry. This two‐step strategy minimizes perturbation during labeling and offers flexible downstream applications.

Bio‐orthogonal click chemistry enables post‐labeling of activity‐based probes (ABPs) with diverse detection tags while preserving probe function and cell permeability. Small reactive handles—typically terminal alkynes or azides—can be incorporated via solid‐phase synthesis (SPPS), unnatural amino acid mutagenesis (e.g., propargyl‐lysine), or metabolic labeling (e.g., azidohomoalanine) [107]. Following target labeling, CuAAC (Cu(I)‐catalyzed azide–alkyne cycloaddition) or SPAAC (strain‐promoted cycloaddition) reactions link these handles to azide‐ or alkyne‐conjugated reporters such as fluorophores or biotin [108]. This modular approach allows multiplexed detection or enrichment from a single probe scaffold. Linkers (PEG_n_, Gly‐Ser) improve tag accessibility and minimize steric hindrance, while cleavable linkers (e.g., disulfide, photocleavable) enable recovery of labeled proteins. Together, these strategies offer a powerful, flexible toolkit for ABP‐based proteomic and imaging applications.

### 9.3 Choosing a Tagging Strategy

The decision between built‐in tags and click handles depends on the biological application and experimental requirements. Built‐in tags are ideal for streamlined workflows and standard detection (e.g., Western blotting). Click handles provide flexibility, improved probe delivery, and compatibility with live‐cell or in situ profiling. Hybrid strategies may be used where, for example, a probe has an HA tag for detection and an alkyne for post‐labeling. Modern studies increasingly favour minimal clickable probes (e.g., Ub–PA) combined with modular tagging reagents to enable customizable, sensitive, and multiplexed readouts.

## 10 Warhead Chemistry and Electrophilic Modifications

### 10.1 Vinyl Methyl Ester and Related Michael Acceptors

**FIGURE 11:**
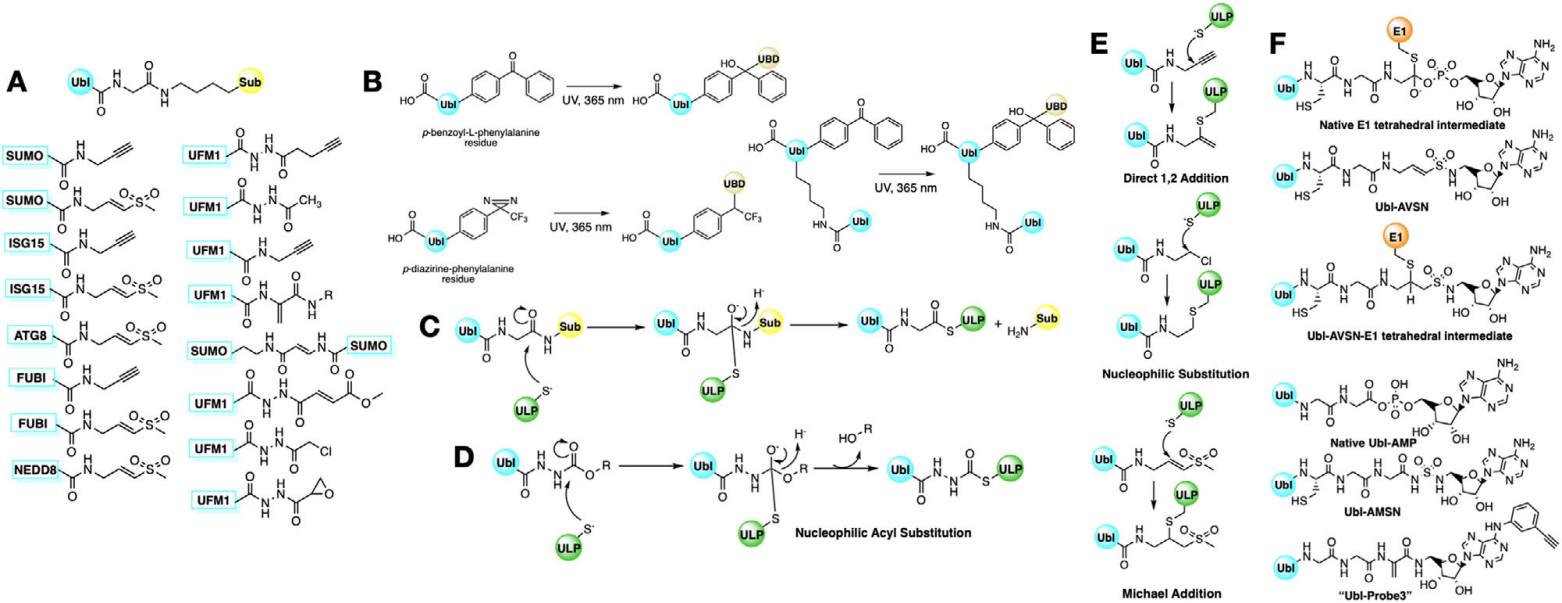
Warhead chemistry of Ubl probes. (A) Ubl probes have been developed so far using different electrophilic warheads. (B) Photoswitchable Ubl probes having *p*‐benzoyl‐L‐phenylalanine (BPA), diazirine group as warhead. (C) Canonical mechanism of ULP‐mediated isopeptide bond hydrolysis. In a nucleophilic acyl substitution reaction, the catalytic cysteine attacks the *C*‐terminal carbonyl of ubiquitin, forming a thioester intermediate, followed by amine release and hydrolysis to regenerate free Ubl. (D) Azapeptide ester warheads, that react with the ULP active‐site cysteine in a manner analogous to natural ubiquitinated substrates, forming a tetrahedral transition state characteristic of acyl transfer reactions, wherein the resulting oxyanion is stabilized by the enzyme's anion hole. (E) Warheads that react with the catalytic cysteine are different from native enzymatic catalysis. These reactions can be categorized into direct (1,2) addition (e.g., PA warhead), Michael addition (e.g., VS warhead), and nucleophilic displacement (e.g., Cl warhead), depending on the nature of the electrophiles. (F) E1 warheads that target E1‐catalytic cysteine (Ubl‐E1 tetrahedral intermediate and Ubl‐adenylate shown for comparison). Ubl‐AVSN mimics Ub‐AMP and covalently traps the E1‐active site. E1 probes that mimics Ubl adenylate complex, such as Ubl‐AMSN, an unreactive analog for Ubl‐AMP. Ubl‐Probe3 reacts similarly to Ubl‐AVSN.

Vinyl methyl ester (VME) warheads represent the classical approach to covalent DUB targeting, functioning through irreversible Michael addition to active site cysteine residues [35]. The VME group provides excellent selectivity for cysteine nucleophiles while maintaining stability under physiological conditions. However, VME probes show limited reactivity toward JAMM family metalloproteases, which lack catalytic cysteine residues [109]. The synthetic installation of VME groups typically proceeds through the coupling of glycine vinyl methyl ester to Ubl *C*‐termini via amide bond formation. This approach requires careful control of reaction conditions to prevent premature hydrolysis or polymerization of the electrophilic warhead. Alternative Michael acceptors such as vinyl sulfone have been developed to modulate reactivity and selectivity profiles (Figure 11A).

### 10.2 Propargylamine Warheads

Propargylamine (PA) warheads offer unique advantages, including broad enzyme targeting capability and reversible covalent bond formation. Unlike VME groups which react via Michael addition PA warheads react with all major DUB families via 1,2‐direct addition (Figure 11E), including JAMM metalloproteases, providing more comprehensive enzyme profiling. The resulting thioether linkage can be cleaved under acidic conditions, enabling recovery of labeled enzymes for further analysis. The installation of PA warheads proceeds through direct aminolysis of Ubl thioesters with propargylamine hydrochloride under basic conditions [19, 20]. This reaction typically achieves high conversion within hours and requires minimal purification. The stability and broad reactivity of PA warheads have made them the preferred choice for many proteomics applications (Figure 11A).

### 10.3 Photoswitchable and Controllable Warheads

The development of Photoswitchable warheads introduces unprecedented control over probe activation and enzyme targeting (Figure 11B). Azobenzene‐containing warheads can be reversibly switched between active and inactive conformations through light irradiation, enabling temporal and spatial control over enzyme labeling [110]. Recent advances include the development of photoactivatable metabolic warheads based on amino‐substituted benzoselenadiazoles. These compounds remain inert until activated by specific wavelengths of light, whereupon they generate highly reactive species capable of covalent protein modification. This approach enables precise spatiotemporal control over probe activation in complex biological systems [111].

### 10.4 Next‐Generation Azapeptide Ester Warheads

Azapeptide ester warheads represent a significant advancement in probe design, offering superior mechanistic mimicry of natural enzyme‐substrate interactions. These warheads incorporate an azaglycine ester at the Ubl's *C*‐terminus, structurally mimicking native ubiquitinated protein substrates. The azapeptide warheads react through an acyl transfer mechanism and engage the enzyme similar to natural enzyme catalysis (Figure 11C, D). The synthesis of azapeptide ester warheads requires specialized chemistry due to the unique azaglycine building block. The electrophilic ester functionality reacts with DUB active site cysteines to form thiocarbamate linkages, providing enhanced stability compared to traditional thioester bonds [58]. Comparative studies demonstrate superior labeling efficiency and enzyme profiling capabilities relative to conventional propargylamine probes.

### 10.5 Warheads Targeting E1 Enzyme

Vinyl sulfonamide warheads, incorporated as Ubl‐AVSN constructs, provide mechanism‐based tools for specifically capturing E1 catalytic cysteines during the thioester formation step (Figure 11F). These probes are designed to trap the incoming E1 cysteine nucleophile in the second half‐reaction of Ubl activation. SUMO‐AVSN and Ubl‐AVSN constructs selectively, covalently, and stably cross‐link to their respective E1 enzymes (SAE1‐UBA2 and UBA1) in a cysteine nucleophile‐dependent manner [112]. Additionally, Ubl–adenylate mimics were developed by replacing the native *C*‐terminal adenylate with a nonreactive acyl‐sulfamide (e.g., SUMO1‐AMSN) [113]. These synthetic adenylate analogs effectively trap transient E1 intermediates and can also function as potent inhibitors of Ubl and E1 activation. Statsyuk and colleagues addressed limitations of the SUMO1‐AVSN probe by designing Ub(Dha)‐based probes for E1 enzymes. By replacing Ub/Ubl's *C*‐terminal glycine with dehydroalanine, they developed “Ub/Ubl‐probe3,” which forms a covalent linkage with the E1 catalytic cysteine via thioether formation [114].

## 11 Future Directions

Despite remarkable progress in the chemical synthesis and application of Ubl‐based activity probes, several pressing challenges and emerging opportunities define the roadmap ahead. A foremost goal is enhancing probe specificity across diverse Ubl families. Most existing electrophilic warheads—such as vinyl sulfones, vinyl amides, or propargylamines—remain broadly reactive, often labeling multiple classes of deconjugating enzymes. Future designs must prioritize chemical discriminability by exploiting subtle differences in enzymatic active sites, such as microenvironmental pKa, secondary shell interactions, or substrate tunnel geometries. Innovations in tunable warhead scaffolds, biselectrophiles, or natural thioester mimetics may offer superior selectivity. A parallel frontier involves developing probes with spatiotemporal control. Photocaged warheads have already enabled light‐dependent Ubl labeling in vitro and in live cells. Expanding such strategies to include redox‐ or enzyme‐responsive triggers could allow dynamic, context‐specific profiling of Ubl pathways. Dual‐reactive probes that combine a covalent warhead and photo‐crosslinker offer a promising strategy to first trap enzymatic targets covalently, followed by spatially resolved capture through UV‐activated crosslinking. However, such approaches demand precise timing, optimized linker flexibility, and minimal phototoxicity.

On the synthesis side, expanding access to full‐length Ubl probes with high functional fidelity remains a key challenge. While SPPS, native chemical ligation (NCL), and ACPL (activated cysteine‐directed protein ligation) offer robust platforms, each comes with trade‐offs in scalability, residue scope, and thioester stability. Emerging strategies such as KAHA ligation and stabilized peptide hydrazides promise enhanced compatibility and synthetic accessibility. For NCL, future improvements will likely center on incorporating β‐ or γ‐thiolated amino acids for ligation at noncysteine sites, followed by desulfurization, or engineering new stable thioester surrogates with minimal side reactions. Chemoenzymatic platforms also hold promise for next‐generation Ubl probe assembly. Sortase‐mediated ligation, E1/E2 trans‐thioesterification, and split inteins offer modular assembly routes for full‐length probes directly in aqueous buffer. Engineering orthogonal E1s, chimeric E2s, or substrate‐fused constructs may streamline Ubl labeling of specific target proteins or enable synthesis‐free capture of transient complexes. Expanding the electrophilic reactivity space beyond cysteine is another key goal. Metalloprotease‐targeting warheads—such as transition‐state mimetics or zinc‐chelating groups—remain underdeveloped but essential for capturing JAMM‐family enzymes. Similarly, integrating fluorogenic handles, singlet‐oxygen generators, or proximity‐labeling groups will enable live‐cell imaging and quantitative profiling at single‐cell resolution.

Finally, the integration of genetic code expansion with chemical synthesis will reshape the next phase of probe development. Site‐specific installation of latent electrophiles or orthogonally protected lysines into Ubls or target proteins enables precise control over conjugation and supports time‐resolved dissection of Ubl signaling in living cells. Hybrid approaches—where synthetically modified Ubls are directly expressed and post‐translationally activated—could streamline workflow and improve cellular delivery. Ultimately, the future of Ubl ABPs lies in merging synthetic ingenuity with systems‐level biology. Probes that are chemically precise, biologically compatible, and dynamically tunable will illuminate Ubl networks in ways not achievable by traditional methods. These tools will not only deepen our mechanistic understanding but may also uncover therapeutic vulnerabilities in diseases driven by dysregulated ubiquitin‐like signaling.

## 12 Conclusions and Outlook

In summary, a diverse arsenal of chemistry‐driven strategies has empowered the development of ABPs for Ubls. Total and semi‐synthesis platforms such as solid‐phase peptide synthesis (SPPS) combined with native chemical ligation (NCL) enable atomic‐level construction of Ubl analogs bearing site‐specific modifications, electrophilic warheads, or functional tags. These synthetic routes offer exquisite structural control and compatibility with diverse probe formats, though often at the expense of scalability and labor intensity. Intein‐mediated protein ligation complements this by producing recombinant Ubls as *C*‐terminal thioesters, which can be selectively functionalized post‐translationally with electrophiles such as vinyl sulfones (VS) or propargylamines (PA). Additionally, click chemistry (e.g., Cu‐catalyzed azide–alkyne cycloaddition) provides modular access to hybrid probes, enabling the coupling of alkyne‐ or azide‐tagged Ubls to biotinylated, fluorescent, or cross‐linkable partners through stable triazole linkages.

Critically, the advent of activated cysteine‐based protein ligation (ACPL) has emerged as a transformative strategy in Ubl probe synthesis. ACPL enables direct conversion of engineered or native cysteine residues at the Ubl *C*‐terminus into chemically reactive intermediates (e.g., dehydroalanine) or conjugated electrophiles without requiring peptide thioesters, intein scaffolds, or protecting group manipulations. By treating cysteinyl Ubls with mild electrophiles or Michael acceptors, researchers can site‐specifically install a variety of warheads including PA, VS, and Dha under near‐physiological conditions. ACPL offers a uniquely accessible, single‐step route to generate folded, functional Ubl probes directly from recombinant proteins, dramatically simplifying the workflow and broadening the method's utility across Ubl classes. Its compatibility with native protein folds, small reagent requirements, and orthogonality to enzymatic or ligation steps position ACPL as a particularly attractive option for scalable and rapid probe development.

Each chemical strategy, SPPS/NCL, intein‐mediated ligation, ACPL, or click chemistry, brings distinct strengths and compromises. SPPS and NCL offer precision and flexibility but require technical expertise. Intein‐based methods achieve native folding and high yields but demand genetic fusions and specialized purification. ACPL stands out for its operational simplicity, one‐pot functionalization, and minimal infrastructure needs, though it currently applies best to terminal cysteine‐modified proteins. Electrophilic warhead design remains a central challenge: achieving a balance between reactivity and selectivity is crucial, with overly reactive warheads risking off‐target modification and less reactive ones capturing only highly active enzymes. Meanwhile, linker design and tag placement (e.g., fluorophores, biotin) must preserve enzymatic recognition while enabling downstream imaging or enrichment. Looking forward, future developments will likely focus on improving regioselectivity, stability, and in vivo compatibility of ABPs. ACPL is particularly well‐suited for evolving these frontiers. Its ability to install diverse chemical functionalities on folded proteins opens the door to caged probes, photoresponsive electrophiles, and conditional conjugates that respond to stimuli or the environment. Moreover, combining ACPL with bioorthogonal labeling techniques, such as strain‐promoted azide–alkyne cycloaddition or tetrazine ligation, could enable the creation of multifunctional or live‐cell compatible Ubl probes. Additionally, integration of ACPL into automated or high‐throughput workflows offers new avenues for library generation and screening applications.

As UBL ABPs continue to mature, their role in translational applications will grow from profiling disease‐associated UBL‐targeting enzymes to validating small‐molecule inhibitors in drug discovery pipelines. The inclusion of ACPL within the chemical biology toolkit ensures that probe development can keep pace with these ambitions: delivering accessible, robust, and modular synthetic solutions for dissecting Ubl enzymatic landscape at unprecedented resolution.

## Conflicts of Interest

The authors declare no competing financial interests.

## Data Availability

Data sharing is not applicable to this article as no new data were created or analyzed in this study.
